# A Light‐Activated Acyl Carrier Protein “Trap” for Intermediate Capture in Type II Iterative Polyketide Biocatalysis

**DOI:** 10.1002/chem.201903662

**Published:** 2019-12-06

**Authors:** Samantha L. Kilgour, David P. A. Kilgour, Panward Prasongpholchai, Peter B. O'Connor, Manuela Tosin

**Affiliations:** ^1^ Department of Chemistry University of Warwick Library Road Coventry CV4 7AL UK; ^2^ Department of Chemistry and Forensics Nottingham Trent University Nottingham NG11 8NS UK

**Keywords:** chemical probes, intermediate capture, iterative polyketide catalysis

## Abstract

A discrete acyl carrier protein (ACP) bearing a photolabile nonhydrolysable carba(dethia) malonyl pantetheine cofactor was chemoenzymatically prepared and utilised for the trapping of biosynthetic polyketide intermediates following light activation. From the in vitro assembly of the polyketides SEK4 and SEK4b, by the type II actinorhodin “minimal” polyketide synthase (PKS), a range of putative ACP‐bound diketides, tetraketides, pentaketides and hexaketides were identified and characterised by FT‐ICR‐MS, providing direct insights on active site accessibility and substrate processing for this enzyme class.

## Graphical abstract

**A novel photoactivatable** acyl carrier protein‐based tool provides novel insights on substrate iterative processing for a model type II iterative polyketide synthase.

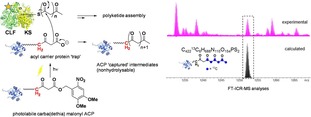

Polyketides constitute a prominent family of structurally and functionally diverse secondary metabolites, comprising renowned pharmaceuticals, agrochemicals and other products of commercial interest.^1^ Their biosynthesis proceeds through multiple decarboxylative Claisen condensation steps, involving acyl carrier protein (ACP) bound malonates and ketosynthase (KS) bound acyl units (Figure 1 and 2 A). A polyketide carbon backbone is assembled and modified, while remaining PKS‐bound, by auxiliary enzymes (ketoreductases, KRs; dehydratases, DHs; and enoylreductases, ERs), until it is eventually released from the PKS (typically by thioesterase (TE) mediated hydrolysis/cyclisation) and further enzymatically elaborated to the mature bioactive product.^2^ PKSs are classified as “modular” or “iterative” and into different types according to their structural organization and modus operandi.^3^

**Figure 1:**
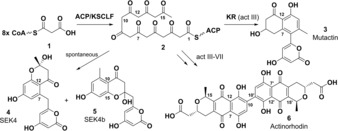
SEK4/SEK4b biosynthesis by the type II actinorhodin (*act*) “minimal system”: malonyl ACP decarboxylative Claisen condensation, driven and controlled by *act* KS‐CLF (Figure 2 A), generates an ACP‐bound octaketide (**2**). In the absence of further enzymatic processing (e.g. by a KR domain), SEK4 and SEK4b (**4** and **5**) are the main products resulting from spontaneous octaketide cyclisation, dehydration and aromatisation. Legend: ACP=acyl carrier protein; KS‐CLF=ketosynthase‐chain length factor; KR=ketoreductase.

**Figure 2:**
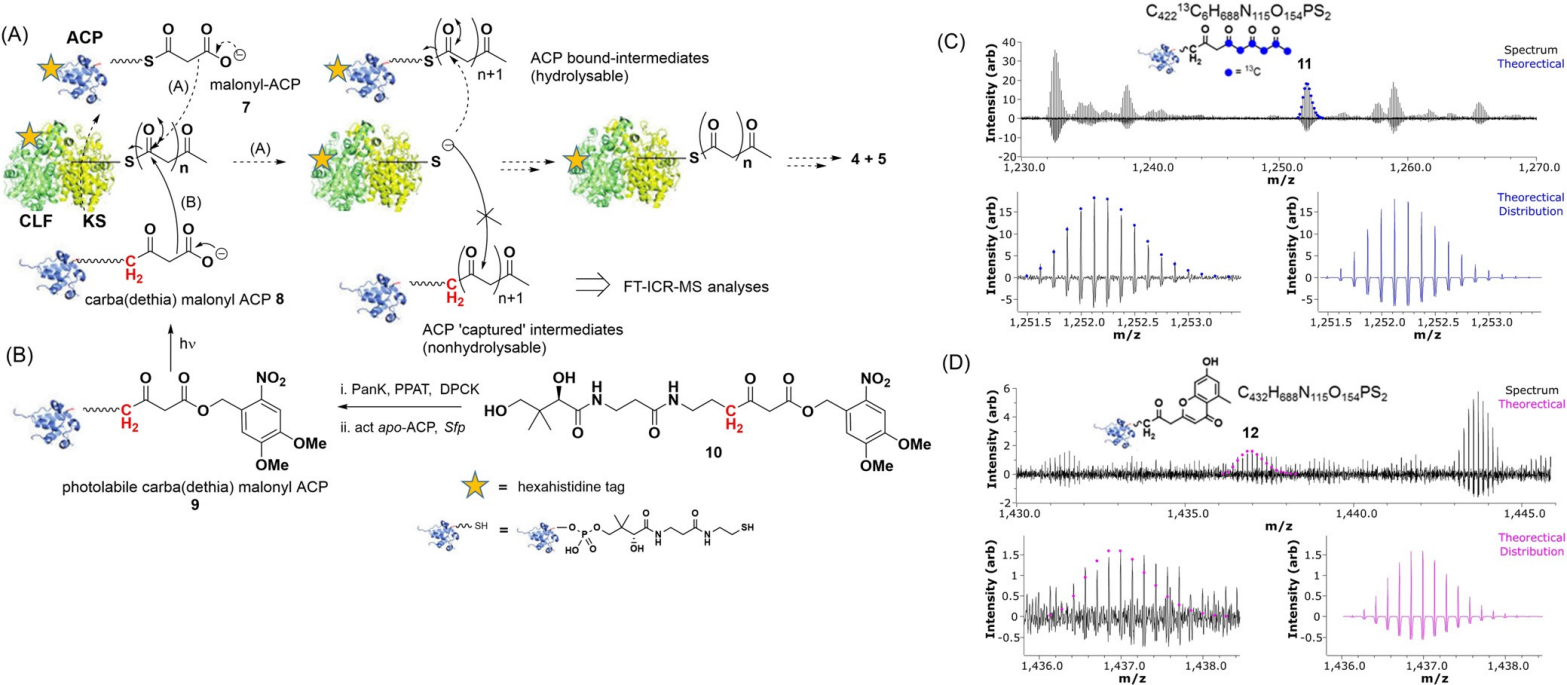
(A) Devised type II PKS intermediate capture by the discrete acyl carrier protein “trap” **8**: this would compete with natural malonyl ACP **7** for polyketide chain extension, leading to accumulation of ACP‐bound nonhydrolysable intermediates. (B) Chemoenzymatic preparation of photolabile carba(dethia) malonyl ACP **9** which, upon irradiation at 365 nm, generates **8**; (C) FT‐ICR‐MS absorption mode detection of a putative nonhydrolysable ACP‐bound tetraketide **11** (^13^C‐labelled, 8+ charged state) in type II minimal *act* PKS assays containing both **8** and ^13^C_3_‐malonyl ACP (SI), with the latter in excess; (D) FT‐ICR‐MS detection of a putative nonhydrolysable ACP‐bound cyclised dehydrated hexaketide species **12** (7^+^ charged state) in similar assays containing both **8** and **7** with the former in excess (for further details, additional detected species and autocorrelation analyses please see the Supporting Information).

Clinically important compounds such as the anticancer agents doxorubicin and daunorubicin are products of type II iterative polyketide synthase (iPKS) biocatalysis.^4^ Iterative PKSs comprise single enzymes (type III), single multi‐domain modules (type I), or discrete enzymes (type II) which repetitively employ the same catalytic activities to assemble and modify polyketide carbon chains. In comparison to modular PKSs, for which the nature of polyketide products is mostly predictable on the basis of module number and composition and can be altered or evolved,^5^ the investigation and the re‐programming of iPKSs remain challenging and underexploited.

Within iPKSs, type II systems are distinctive biomolecular factories: they are found prevalently in Gram positive *Actinomycetes* 4 and are made of discrete proteins acting in a concerted manner to ultimately generate complex aromatic metabolites, including tetracyclines, anthracyclines, benzoisochromanequinones, tetracenomycins, aureolic acids, angucyclines and pentagular polyphenols. Type II PKSs closely resemble type II fatty acid synthases (FASs) in their essential mechanisms of substrate processing, however they differ in terms of intermediate nature and substrate binding modes adopted by their essential ACP components.^6^ Over time, the complex nature of protein–protein and protein–substrate interactions, as well as the fast kinetics of product assembly presented by these enzymes, have been the object of intense scrutiny.^7^ For these studies model type II “minimal” PKS systems have often been used. In this work, the actinorhodin minimal system has been our model system of choice.

A type II “minimal” PKS is constituted by a heterodimeric ketosynthase (KS)—chain length control (CLF) domain,^8^ which catalyses and controls polyketide chain initiation and elongation; and by a discrete acyl carrier protein (ACP). This ACP delivers malonyl building blocks and intermediates to the KS‐CLF complex to construct a polyketone chain via the 4′‐phosphopantetheine (PPant) cofactor (Figures 1 and 2 A).^9^ A malonyl Coenzyme A: ACP transacylase (MCAT) normally provides malonyl extender units to discrete type II ACPs, however this is not strictly required for a minimal system to function as type II ACPs can self‐malonylate.^10^ In the absence of the ketoreductase *actIII* the postulated ACP‐bound octaketide (**2**) spontaneously folds to afford shunt products: in the case of the aromatic antibiotic actinorhodin, these are the octaketides SEK4 and SEK4b (**4** and **5** respectively, Figure 1).^8^, ^10^ In the presence of the ketoreductase *actIII* the postulated ACP‐bound octaketide (**2**) is converted to mutactin (**3**, Figure 1),^11^ whereas the combined action of *actIII–actVII* (act ketoreductases, aromatase, cyclase and oxidases) ultimately convert **2** to actinorhodin (**6**).^12^

For type II PKSs, malonyl‐CoA remain the only elongating unit known to date, whereas a variety of acyl building blocks (e.g., acetate, propionate, (iso)butyrate, benzoate…) can be used to prime these enzymes, and, together with post‐PKS tailoring enzymes (e.g., cyclases, oxidases, aromatases and methyltransferases), contribute to the structural variation of type II PKS products.^4^ In vitro reconstitution of enzyme activity^8^, ^10^ and in vivo genetic manipulation^13^ have proved crucial in gathering the first insights into the determinants of enzyme priming and chain length control. More recent biophysical studies (e.g., X‐ray crystallography^14^ and NMR^15^) of type II PKS proteins have provided more in‐depth knowledge on protein/ substrate recognition and productive conformations. Some of these investigations have relied on the enzymatic loading of synthetically prepared intermediate mimics of ACPs, as the natural ACP‐bound intermediates are intrinsically unstable and highly reactive.^15^, ^16^ The general inability of directly monitoring iterative intermediate formation and processing in real time constitutes a significant hurdle in gathering new knowledge of enzyme kinetics and protein‐substrate interactions required to devise novel synthetic biology.^17^ In our labs we have established a chemical ‘chain termination“ methodology aimed at the capture of transient polyketide biosynthetic intermediates in vitro^18^ and in vivo.^19^, ^20^ The method is based on the use of small molecule probes that are nonhydrolysable mimics of ACP‐bound malonate units. In competition with these last, the probes react with enzyme‐bound biosynthetic intermediates to off‐load them in a readily available and stable form for LC‐MS characterisation. More recently we also reported the development of nonhydrolysable mimics of PCP (peptidyl carrier protein)‐bound amino acids for the investigation of nonribosomal peptide assembly.^21^

Herein we sought to extend the scope of our methodology to develop protein‐based tools aimed at intermediate capture in a more stringent fashion. In particular, we envisaged that an acyl carrier protein, modified with a nonhydrolysable mimic of malonyl pantetheine, may act as an intermediate “trap” for biosynthetic intermediates involved in type II PKS assembly, in competition with the natural malonyl ACP **7** (Figure 2). In order to explore this and gather novel insights into the *act* type II PKS minimal system, a photoactivatable acyl carrier protein **9** was chemoenzymatically prepared according to Schemes S1 and S2 in the Supporting Information. The 4,5‐dimethoxy‐2‐nitrobenzyl (DMNB) photolabile group^22^ was chosen as a means to protect the pseudo‐malonate moiety of carba(dethia) malonyl ACP **8,** due to its relative ease of synthetic incorporation and controllable removal at a non‐protein damaging wavelength (365 nm).

From commercially available d‐pantothenic acid the photolabile pantetheine derivative **10** was synthesised in 6 steps (Scheme S1) and employed as substrate for the recombinant *E. coli* enzymes PanK, PPAT and DPCK,^23^ to generate the corresponding coenzyme A photolabile derivative (Scheme S2). Upon incubation of the latter with recombinant *act* apo‐ACP and the phosphopantetheinyl transferase Sfp,^24^ the desired photolabile ACP probe **9** was obtained (Figure 2 B). Light‐activation of **9** was tested in the absence and in the presence of the *act* KS‐CLF, employing either a KiloArc Broadband Arc Lamp or in an in‐house built light box containing a circular 22 W UVA lamp^22^ (Supporting Information). While isolated **9** could be deprotected to **8** within a 4 hour irradiation period (Figure S2), negligible deprotection of **9** took place in the presence of the *act* KS‐CLF (Figure S3). Enzymatic assays for the production of SEK4 and SEK4b by hexahistidine‐tagged *act* ACP and KS‐CLF were set up as previously reported,^10^, ^25^ adding the pre‐photolysed and untagged ACP **8** at different times and variable concentrations (Table S1). Enzymatic assay filtration through Ni‐NTA agarose beads was carried out in order to selectively isolate any species deriving from the “unnatural” ACP **8**. The samples recovered from this operation were concentrated and buffer‐exchanged ahead of direct infusion into an FT‐ICR‐MS spectrometer (Supporting Information). The outcome of these experiments is illustrated in Figure 2 and detailed in the Supporting Information (Table S1).

In selected samples, putative ACP‐bound nonhydrolysable intermediates, including di, tetra, penta and hexaketide species (including mono‐ and di‐dehydro species) were identified and characterised by HR‐MS analysis of protein‐charged states ranging from 11+ to 6+; these species were absent in control samples (Supporting Information). Also, in assays of KS‐CLF with ^13^C_3_‐malonyl ACP (instead of **7**) and **8**, putative ACP‐bound nonhydrolysable species bearing an even number of ^13^C atoms were observed (Figure 2 C and Supporting Information), consistent with their expected polyketide nature. Overall, in the analyses of KS‐CLF assays in the presence of **8**, carba(dethia) acetyl‐ACP was the most abundant species, whereas the putative captured intermediates were present in low abundance. To characterise further these species without additional sample manipulation, the 4′‐phosphopantetheine (PPant) ejection assay^26^ was attempted directly on the heterogeneous samples infused into the FT‐ICR‐MS, however this did not lead to small molecule detection. The 4′‐PPant ejection assay employs collisionally activated dissociation (CAD) to preferentially cleave the 4′PPant ion. However, we cannot exclude CAD possibly fragmenting the enzyme‐bound polyketide species in the conditions employed to analyse such complex mixtures. In order to improve the confidence with which we identified low abundance putative ACP‐bound species, we used autocorrelation to identify periodic patterns in data. The result of the isotopic distribution of the ion at the right charge state can indeed support the identification of signals close to the noise threshold, as demonstrated by Palmblad et al.^27^ The results of this approach applied to our samples supported the assignment of the manually identified putative species (see Supporting Information).

Amongst the identified species, ACP‐bound diketide and tetraketide species were most abundantly detected in samples deriving from the simultaneous addition of both **8** and **7**, whereas a putative di‐dehydro hexaketide (Figure S9) was the most commonly observed product, in a variety of conditions (Table S1). Parallel experiments conducted using photolabile *N*‐acetylcysteamine‐based chain termination probes,^22^ in the in vitro assembly of SEK4 and SEK4b, did not lead to any off‐loaded putative intermediates (data not shown), leading us to postulate that the ACP probe interacts more efficiently with the minimal system.

The assembly of actinorhodin by the *act* PKS, as well as that of related products and other type II PKS‐derived metabolites, has been the object of intensive scrutiny and still holds a number of unresolved questions, including several concerning intermediate sequestration and stabilisation and protein‐protein interactions.^15^ Herein we have shown that, through the use of a chemoenzymatically generated malonyl ACP nonhydrolysable mimic (**8**), direct evidence of novel ACP‐bound polyketone species involved in type II PKS assembly can be obtained. These species, in contrast to others previously reported,^28^ are nonhydrolysable from the carrier protein, hence they should constitute useful chemical biology tools for mechanistic and structural investigations.

The ability of **8** to intercept putative biosynthetic intermediates in vitro, such as those herein presented, and, conversely, the inability of *N*‐acetylcysteamine based probes to do so (data not shown), support that, for the type II *act* minimal system, the KS‐CLF active site is mostly accessible to ACP‐bound substrates rather than free species. The varied nature of putative ACP‐bound intermediates observed in our experiments, including in those where the unnatural pseudo‐malonyl ACP **8** was present in defect to the natural malonyl ACP substrate **7**, suggests dynamic interactions between the KS‐CLF and the ACP, with proteins that can interchange,^29^ and protein–protein interactions efficiently guiding ACP‐bound substrates into the KS active site for processing.^30^ The putative captured ACP‐bound species detected in these experiments may possibly reflect the kinetics of carbon chain assembly and folding, with specific steps, for example, diketide and tetraketide formation, relatively slow in comparison to others. This would be in agreement with crystallographic studies of *act* KS‐CLF “caught in action” with a diketide and a tetraketide species bound to its cysteine active site.^14^ Further work will be required to corroborate the preliminary insights gathered by our experiments. Nonetheless, the ACP probe **8** herein prepared and evaluated constitutes a rare example of protein “trap” for biosynthetic species^31^ and the first for polyketide synthases. Its use in conjunction with advanced FT‐ICR‐MS analyses and data analysis tools represents a new promising approach for the study of challenging biosynthetic enzymes that make use of dynamic carrier proteins, including fatty acid synthases and nonribosomal peptide synthetases.

## Experimental Section

The chemoenzymatic preparation of **9**, its photolysis to **8** and use of **8** in enzymatic assays generating SEK4 and SEK4b, as well as FT‐ICR‐MS analyses of putative captured enzyme‐bound intermediates, are reported in the Supporting Information.

## Conflict of interest

The authors declare no conflict of interest.

## Supporting information

As a service to our authors and readers, this journal provides supporting information supplied by the authors. Such materials are peer reviewed and may be re‐organized for online delivery, but are not copy‐edited or typeset. Technical support issues arising from supporting information (other than missing files) should be addressed to the authors.

Supplementary
