# Supplementary material for: A Light‐Activated Acyl Carrier Protein “Trap” for Intermediate Capture in Type II Iterative Polyketide Biocatalysis
Source: Chemistry. 2019 Dec 6;25(72):16515–8. doi: 10.1002/chem.201903662 (PMC6972679; doi:10.1002/chem.201903662)
Supplement: Supplementary file 1 — Supplementary [file CHEM-25-16515-s001.pdf]

CHEMISTRY
A European Journal

Supporting Information

A Light-Activated Acyl Carrier Protein "Trap" for Intermediate Capture in Type II Iterative Polyketide Biocatalysis

Samantha L. Kilgour,[a] David P. A. Kilgour,[b] Panward Prasongpholchai,[a]
Peter B. O'Connor,*[a] and Manuela Tosin*[a]

chem_201903662_sm_miscellaneous_information.pdf

Contents

[1 PREPARATION OF THE PHOTOACTIVATABLE ACYL CARRIER PROTEIN 9 — 2]

1.1 GENERAL SYNTHETIC METHODS — 2
1.2 SYNTHESIS OF 4,5-DIMETHOXY-2-NITROBENZYL (*R*)-6-(3-(2,4-DIHYDROXY-3,3-DIMETHYLBUTANAMIDO)PROPANAMIDO)-3-OXOHEXANOATE (10) — 3
1.3 CHEMOENZYMATIC PREPARATION OF 9 — 9

2 PHOTOLYSIS OF 9 TO GENERATE CARBA(DETHIA) MALONYL ACP (8) — 12

3 IN VITRO INTERMEDIATE CAPTURE FROM THE TYPE II ACT MINIMAL PKS VIA CARBA(DETHIA) MALONYL ACP (8) — 15

3.1 GENERAL METHODS — 15
3.1.1 EXPRESSION AND PURIFICATION OF HEXAHISTIDINE-TAGGED PROTEINS IN *E. COLI* (*ACT* APOACP, SFP, PANK, PPAT AND DPCK) — 15
3.1.2 EXPRESSION AND PURIFICATION OF *ACT* KS-CLF IN *S. COELICOLOR* — 16
3.1.3 THROMBIN CLEAVAGE OF HEXAHISTIDINE-TAG FROM *ACT* ACP — 17
3.1.4 PHOSPHOPANTETHEINYLATION OF ACT-*APO*ACPS — 17
3.1.5 PRIMING OF *ACT* KS-CLF — 18
3.1.6 RECONSTITUTION OF ENZYMATIC ACTIVITY FOR THE *ACT* PKS MINIMAL SYSTEM — 18
3.1.7 INTERMEDIATE CAPTURE FROM THE *ACT* PKS MINIMAL SYSTEM *VIA* THE ACP PROBE 8 AND FT-ICR-MS CHARACTERIZATION — 19

4 NMR SPECTRA — 35

4.1 ^{1}H- AND ^{13}C- NMRS OF COMPOUND 14 — 35
4.2 ^{1}H- AND ^{13}C- NMRS OF COMPOUND 15 — 36
4.3 ^{1}H- AND ^{13}C- NMRS OF COMPOUND 16 — 37
4.4 ^{1}H- AND ^{13}C- NMRS OF COMPOUND 10 — 38
4.5 ^{1}H- AND ^{13}C- NMRS OF COMPOUND 17 — 39

5 REFERENCES — 40

1 Preparation of the photoactivatable acyl carrier protein 9

1.1 General synthetic methods

Unless specified otherwise, chemicals were purchased from Sigma Aldrich, Fisher Scientific, Carbosynth and Alfa Aesar and were used without further purification. Anhydrous dichloromethane and toluene were purchased from VWR International (AR grade) and dried using solvent towers. Anhydrous ethyl acetate, isopropanol, butanol, dimethyl sulfoxide (DMSO) and pyridine were purchased from Fisher Scientific. Reagent grade dichloromethane (DCM), ethyl acetate (EtOAc), methanol (MeOH), acetonitrile (MeCN), cyclohexane, butanol, chloroform ($CHCl_3$) and tetrahydrofuran (THF) were purchased from Fisher Scientific.

Analytical thin-layer chromatography (TLC) was performed on aluminium sheets precoated with silica gel 60 (F_{254}, Merck) and visualized under ultra-violet light (short and long-wave) using potassium permanganate ($KMnO_4$) or vanillin stains. Silica gel for flash chromatography was purchased from Sigma Aldrich (Tech Grade, pore size 60 Å, 230-400 mesh).

^{1}H and ^{13}C NMR spectra were recorded in d_4-MeOD, $CDCl_3$ or D_2O on the following Bruker Avance instruments: DPX-300 300 MHz, DPX-400 400 MHz, DRX-500 500 MHz, AV III-500 HD 500 MHz, AV-600 600 MHz or AV-700 700 MHz.

High-resolution mass spectra (HRMS) of newly made compounds were obtained using electrospray ionization (ESI) on a MaXis UHR-TOF (Bruker Daltonics) or on Bruker MaXis (ESI-HR-MS).

Compounds were purified by semi-preparative HPLC on a Phenomenex Synergi™ Polar RP 80 Å (250 x 10.0 mm, 4µm) column. The mobile phase consisted of a gradient of water (solvent A) and acetonitrile (solvent B) both HPLC graded and containing 0.1 % trifluoroacetic acid- at a flow rate of 2.5 mL/min, with UV detection at 210, 254 and 280 nm.

Time (mins)	% of solvent A	% of solvent B	Flow rate (ml/min)	
			Semi-preparative	Preparative
0	95	5	2.5	20
30	5	95	2.5	20
35	5	95	2.5	20
40	95	5	2.5	20

1.2 Synthesis of 4,5-dimethoxy-2-nitrobenzyl (*R*)-6-(3-(2,4-dihydroxy-3,3-dimethylbutanamido)propanamido)-3-oxohexanoate (10)

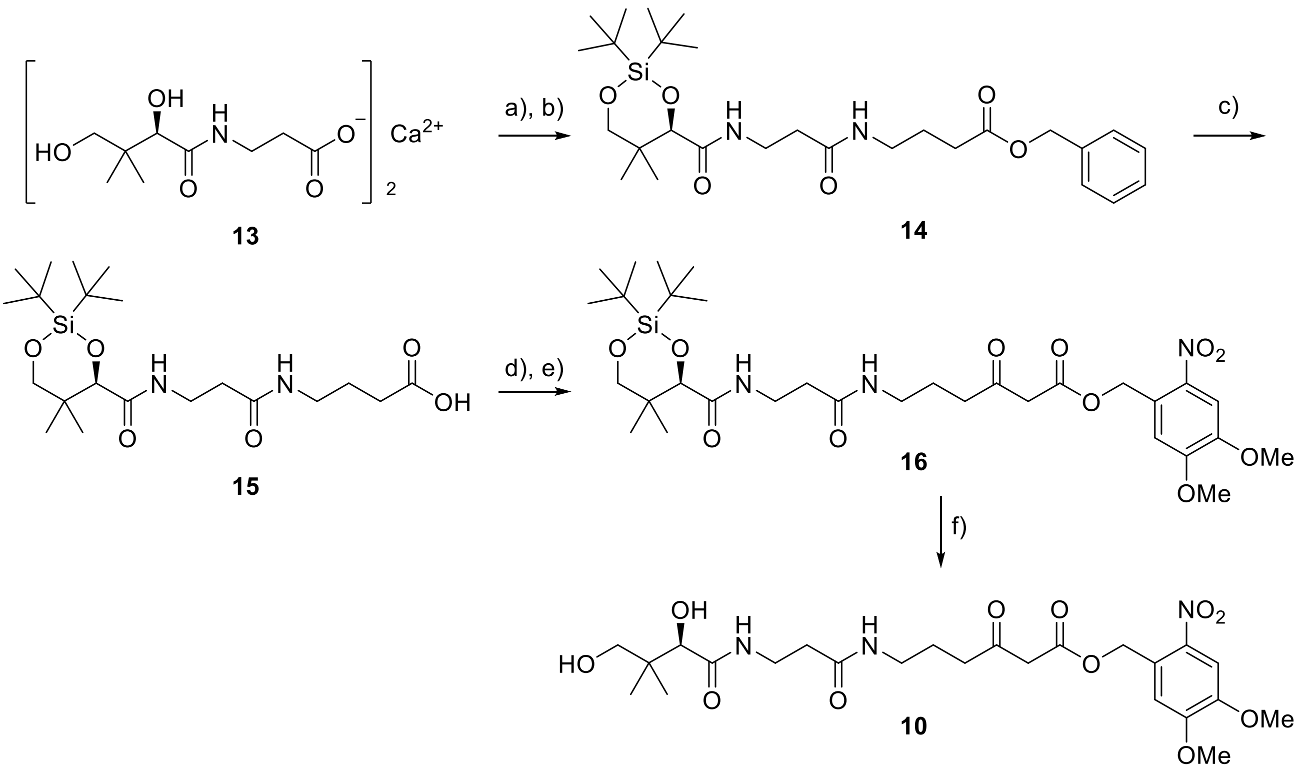

Scheme 1S: Preparation of **10.** a) $(CF_3SO_3)_2Si[C(CH_3)_3]_2$, 2,6-lutidine, THF, room temperature (RT), 18 h; b) γ-aminobutyric acid benzyl ester *p*-tosylate, DIPEA, HATU, THF, RT, 18 h, 39% over two steps; c) Pd/C, $H_{2(g)}$, RT, EtOAc, 2 h, 84%; d) Meldrum's acid, DMAP, EDC.HCl, THF, RT, 18 h; e) 4,5-dimethoxy-2-nitrobenzyl alcohol, toluene, reflux, 8 h, 58% over two steps; f) HF/pyridine, THF, RT, 2 min, 90%.

Benzyl (*R*)-4-(3-(2,2-di-*tert*-butyl-5,5-dimethyl-1,3,2-dioxasilinane-4-carboxamido)propanamido)butanoate (14)

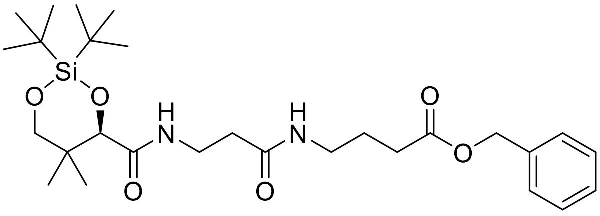

14

To a solution of D-pantothenic acid hemicalcium salt **13** (2.00 g, 8.4 mmol) in dry THF (10 mL) under argon atmosphere 2,6-lutidine (2.93 mL, 25.2 mmol) was added. The solution was cooled to 0°C then di-*tert*-butylsilyl bis(trifluoromethanesulfonate) (3.60 mL, 11.1 mmol) was added dropwise. The reaction mixture was stirred at room temperature for 18 hours. The solvent was removed *in vacuo* and the resulting residue was dissolved in EtOAc (15 mL) and washed with water (15 mL). The organic layer was dried over $MgSO_4$, filtered and concentrated to afford a crude (*R*)-3-(2,2-di-*tert*-butyl-5,5-dimethyl-1,3,2-dioxasilinane-4-carboxamido)propanoic acid (lutidine salt) as a yellow oil (3.29 g). A portion of it was purified by semi-preparative HPLC for detailed NMR characterisation; R_t = 20.3 mins.

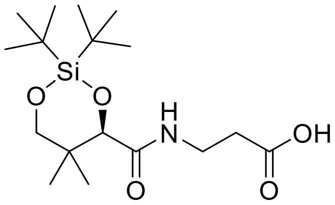

^{1}H-NMR (400 MHz, $CDCl_3$) δ 7.41 (br t, 1H, *J* 5.5 Hz, N*H*), 4.44 (s, 1H, C*H*), 4.04 (d, 1H, *J* 11.5 Hz, C*H*), 3.69-3.64 (m, 1H, C*H*), 3.51 (d, 1H, *J* 11.5 Hz, C*H*), 3.48 (m, 1H, C*H*), 2.61 (t, 2H, *J* 5.5, 6.0 Hz, C*H*$_2$), 1.09 (s, 9H, C*H*$_3$), 1.08 (s, 9H, C*H*$_3$), 1.05 (s, 3H, C*H*$_3$), 1.04 (s, 3H, C*H*$_3$); **HRMS**: *m/z* $[M + Na]^+$, found: 382.2033, calculated: 382.2020.

To a solution of the crude intermediate above (1.51 g) and γ-aminobutyric acid benzyl ester p-tosylate (970 mg, 2.7 mmol) in dry THF (16 mL) under argon atmosphere, *N*,*N*-diisopropylethylamine (DIPEA, 1.50 mL, 8.6 mmol) was added. The reaction mixture was cooled to 0°C for 10 minutes and *O*-(7-azabenzotriazol-1-yl)-*N,N,N′,N′*-tetramethyluronium hexafluorophosphate (HATU, 2.08 g,

5.5 mmol) was added. The reaction was stirred at 0°C for 30 minutes and then allowed to warm to room temperature and stirred for further 18 hours. The solvent was removed *in vacuo* and the resulting residue dissolved in EtOAc (100 mL) and washed thrice with water (100 mL). The organic layer was dried over $MgSO_4$, filtered and concentrated to afford a crude residue, which was purified *via* flash chromatography with gradient elution to afford the desired product **14** (864 mg, 39% over two steps). R_f = 0.4 in 3: 2 EtOAc : DCM containing 1% Et_3N.

^{1}H-NMR (500 MHz, $CDCl_3$) δ 7.37-7.34 (m, 6H, N*H* and Ar*H*), 5.94 (br t, 1H, *J* 5.0 Hz, N*H*), 5.11 (s, 2H, C*H*$_2$), 4.41 (s, 1H, C*H*), 4.03 (d, 1H, *J* 11.5 Hz, C*H*), 3.67-3.59 (m, 1H, C*H*), 3.49 (d, 1H, *J* 11.5 Hz, C*H*), 3.50-3.48 (m, 1H, C*H*), 3.32-3.22 (m, 2H, C*H*$_2$), 2.41-2.38 (t, 2H, *J* 6.0 Hz, C*H*$_2$), 2.40-2.37 (t, 2H, *J* 7.0 Hz, C*H*$_2$), 1.87 (dt, 2H, *J* 6.5, 7.5 Hz, C*H*$_2$), 1.08 (s, 9H, C*H*$_3$), 1.07 (s, 9H, C*H*$_3$), 1.03 (s, 3H, C*H*$_3$), 1.02 (s, 3H, C*H*$_3$); **^{13}C-NMR** (125 MHz, $CDCl_3$) δ 173.3 (*C*OO), 171.6 (*C*ONH), 171.0 (*C*ONH), 135.9 (Ar*C*), 128.8 (Ar*C*H), 128.5 (Ar*C*H), 128.4 (Ar*C*H), 81.5(O*C*H), 75.6 (O*C*H$_2$), 66.6 (Ar*C*H$_2$), 39.1 (*C*H$_2$), 37.5 (*C*H$_2$), 36.2 (*C*), 34.8 (*C*H$_2$), 31.9 (*C*H$_2$), 28.8 (*C*H$_3$), 27.7 (*C*H$_3$), 24.7 (*C*H$_2$), 23.4 (*C*H$_3$), 20.4 (Si*C*), 19.2 (*C*H$_3$); **HRMS**: *m/z* $[M + H]^+$, found: 535.3201, calculated: 535.3198.

(*R*)-4-(3-(2,2-di-*tert*-butyl-5,5-dimethyl-1,3,2-dioxasilinane-4-carboxamido)propanamido)butanoic acid (15)

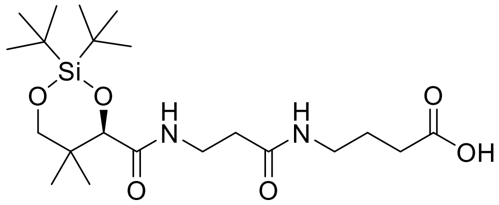

15

A solution of **14** (50 mg, 90 µmol) and palladium on carbon (10 wt. %, 50 mg, 470 µmol) was stirred in dry EtOAc (10 mL) under H_2 atmosphere for 2 hours. The reaction mixture was filtered through Celite and the solvent removed *in vacuo* affording **15** as a white solid (35 mg, 84%).

^{1}H-NMR (500 MHz, $CDCl_3$) δ 7.41 (br t, 1H, *J* 6.0 Hz, N*H*), 6.75 (t, 1H, *J* 5.5 Hz, N*H*), 4.39 (s, 1H, C*H*), 4.00 (d, 1H, *J* 11.5 Hz, C*H*), 3.62-3.56 (m, 1H, C*H*), 3.53-3.48 (m, 1H, C*H*), 3.47 (d, 1H, *J* 11.5 Hz, C*H*), 3.30-3.21 (m, 2H, C*H*$_2$), 2.49-2.39 (m, 2H, C*H*$_2$), 2.34 (t, 2H, *J* 7.0 Hz, C*H*$_2$), 1.80 (quin, 2H, *J* 7.0 Hz, C*H*$_2$), 1.06 (s, 9H, C*H*$_3$), 1.05 (s, 9H, C*H*$_3$), 1.01 (s, 3H, C*H*$_3$), 0.94 (s, 3H, C*H*$_3$); **^{13}C-NMR** (125 MHz, $CDCl_3$) δ 176.6 (*C*OO), 172.2 (*C*ONH), 171.6 (*C*ONH), 81.4 (O*C*H), 75.4 (O*C*H$_2$), 39.1 (*C*H$_2$), 37.5 (*C*H$_2$),

36.0 (CH_2), 31.5 (CH_2), 28.7 (CH_3), 27.8 (CH_3), 24.6 (CH_2), 23.5 (C), 23.7 (CH_3), 20.4 (SiC), 18.9 (CH_3). HRMS: *m/z* $[M - H]^-$, found: 443.2653, calculated: 443.2583.

4,5-dimethoxy-2-nitrobenzyl (*R*)-6-(3-(2,2-di-*tert*-butyl-5,5-dimethyl-1,3,2-dioxasilinane-4-carboxamido)propanamido)-3-oxohexanoate (16)

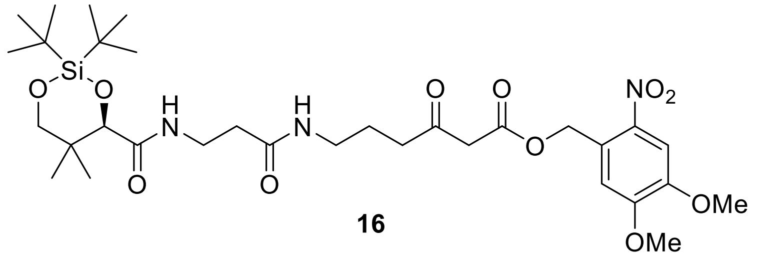

16

To a solution of **15** (603 mg, 1.4 mmol) and Meldrum's acid (216 mg, 1.5 mmol) in dry THF (30 mL) under argon atmosphere was added *N,N*-dimethylpyridin-4-amine (DMAP, 208 mg, 1.7 mmol). After stirring for 10 minutes, 1-ethyl-3-(3-dimethylaminopropyl) carbodiimide hydrochloride (EDC.HCl, 232 mg, 1.2 mmol) was added and the reaction mixture was stirred at room temperature for 18 hours. The solvent was removed *in vacuo* and the resulting residue was dissolved in EtOAc (50 mL), washed once with 1M HCl (50 mL) and washed twice with water (50 mL). The organic layer was dried over $MgSO_4$, filtered and concentrated to afford a crude (*R*)-2,2-di-*tert*-butyl-*N*-(3-((4-(2,2-dimethyl-4,6-dioxo-1,3-dioxan-5-ylidene)-4-hydroxybutyl)amino)-3-oxopropyl)-5,5-dimethyl-1,3,2-dioxasilinane-4-carboxamide (below) as a yellow oil (35 mg); R_f = 0.4 in 4 : 1 EtOAc : MeOH containing 1% Et_3N.

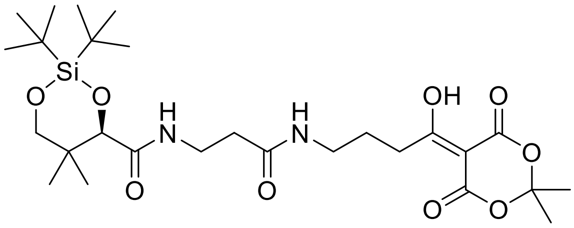

^{1}H-NMR (400 MHz, $CDCl_3$) δ 7.33 (br t, 1H, *J* 6.0 Hz, N*H*), 7.08 (br m, 1H, N*H*), 4.38 (s, 1H, C*H*), 4.00 (d, 1H, *J* 11.5 Hz, C*H*), 3.63-3.58 (m, 1H, C*H*), 3.47 (d, 1H, *J* 11.5 Hz, C*H*), 3.45-3.40 (m, 1H, C*H*), 3.20-3.15 (m, 2H, C*H*$_2$), 2.84 (t, 2H, *J* 7.0 Hz, C*H*$_2$), 2.47-2.37 (m, 2H, C*H*$_2$), 1.75 (m, 2H, C*H*$_2$), 1.61 (s, 6H,

OCCH_3), 1.06 (s, 9H, CH_3), 1.06 (s, 9H, CH_3), 1.04 (s, 3H, CH_3), 0.98 (s, 3H, CH_3); HRMS: *m/z* $[M + Na]^+$, found: 593.2870, calculated: 593.2865.

A solution of the Meldrum's acid adduct above (190 mg) and 4,5-dimethoxy-2-nitrobenzyl alcohol (213 mg, 0.99 mmol) in dry toluene (10 mL) was heated under reflux for 8 hours. The solvent was removed *in vacuo* to afford a crude residue which was purified by preparative HPLC to give **16** as a yellow solid (127 mg, 58%); R_t = 20.3 mins.

^{1}H-NMR (400 MHz, $CDCl_3$) δ 7.72 (s, 1H, Ar*H*), 7.35 (br t, 1H, *J* 6.0 Hz, N*H*), 7.13 (s, 1H, Ar*H*), 6.14 (br s, 1H, N*H*), 5.58 (s, 2H, ArCH_2), 4.41 (s, 1H, C*H*), 4.05 (s, 3H, OCH_3), 4.02 (d, 1H, *J* 11.5 Hz, C*H*), 3.96 (s, 3H, OCH_3), 3.67-3.63 (m, 1H, C*H*), 3.58 (s, 2H, CH_2), 3.51-3.48 (m, 1H, C*H*), 3.50 (d, 1H, *J* 11.5 Hz, C*H*), 3.30-3.19 (m, 2H, CH_2), 2.61 (t, 2H, *J* 7.0 Hz, CH_2), 2.45-2.42 (m, 2H, CH_2), 1.83-1.77 (dt, 2H, *J* 7.0 Hz, CH_2), 1.08 (s, 9H, CH_3), 1.07 (s, 9H, CH_3), 1.03 (s, 3H, CH_3), 1.02 (s, 3H, CH_3); **^{13}C-NMR** (175 MHz, CDCl3): δ 202.3 (*C*O), 171.5 (*C*ONH), 171.2 (*C*ONH), 166.7 (*C*OO), 154.0 (Ar*C*OCH_3), 148.4 (Ar*C*OCH_3), 139.7 (Ar*C*NO_2), 126.9 (Ar*C*), 110.6 (ArCH), 108.3 (Ar*C*H), 81.4 (O*C*H), 75.5 (O*C*H_2), 64.2 (Ar*C*H_2), 56.9 (O*C*H_3), 56.5 (O*C*H_3), 49.2 (CO*C*H_2CO), 40.5 (*C*H_2), 38.6 (*C*H_2), 36.0 (*C*H_2), 34.8 (*C*H_2), 28.7 (*C*H_3), 27.6 (*C*H_3), 23.4 (*C*H_2), 23.4 (*C*H_3), 20.3 (*C*), 18.9 (Si*C*), 18.9 (*C*H_3); **HRMS**: *m/z* $[M + Na]^+$, found: 704.3188, calculated: 704.3185.

4,5-dimethoxy-2-nitrobenzyl (*R*)-6-(3-(2,4-dihydroxy-3,3-dimethylbutanamido)propanamido)-3-oxohexanoate (10)

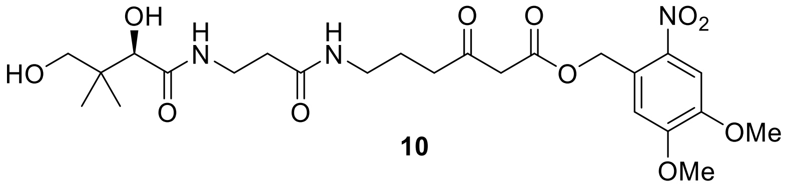

10

A solution of **16** (79 mg, 0.12 mmol) and hydrogen fluoride pyridine (52 µl, 0.58 mmol) in dry THF (0.5 mL) was stirred for 2 mins at room temperature. The solvent and hydrogen fluoride pyridine

were removed *in vacuo* to afford crude product which was purified by preparative HPLC to give a yellow solid **22** (58 mg, 90%); R_t = 18 mins.

^{1}H-NMR (500 MHz, $CDCl_3$): δ 7.71 (s, 1H, Ar*H*), 7.39 (br t, 1H, *J* 6.0 Hz, N*H*), 7.11 (s, 1H, Ar*H*), 6.41 (br t, 1H, *J* 5.5 Hz, N*H*), 5.56 (s, 2H, ArC*H*$_2$), 4.04 (s, 3H, OC*H*$_3$), 3.98 (s, 1H, OC*H*), 3.95 (s, 3H, OC*H*$_3$), 3.60 (s, 2H, C*H*$_2$), 3.58-3.52 (m, 2H, C*H*$_2$), 3.47 (s, 2H, HOC*H*$_2$), 3.31-3.18 (m, 1H, C*H*$_2$), 2.62 (t, 2H, *J* 7.0 Hz, C*H*$_2$), 2.42 (t, 2H, *J* 6.0 Hz, C*H*$_2$), 1.82 (dt, 2H, *J* 7.0 Hz, C*H*$_2$), 0.99 (s, 3H, C*H*$_3$), 0.90 (s, 3H, C*H*$_3$); **^{13}C-NMR** (125 MHz, $CDCl_3$) δ 202.7 (*C*O), 173.8 (*C*ONH), 171.7 (*C*ONH), 176.0 (*C*OO), 153.8 (Ar*C*OMe), 148.6 (Ar*C*OMe), 139.9 (Ar*C*NO$_2$), 126.6 (Ar*C*), 110.9 (Ar*C*H), 108.4 (Ar*C*H), 77.8 (O*C*H), 71.1 (O*C*H$_2$), 64.4 (Ar*C*H$_2$), 56.8 (O*C*H$_3$), 56.6 (O*C*H$_3$), 49.3 (*C*H$_2$), 40.6 (*C*H$_2$), 39.5 (*C*), 38.8 (*C*H$_2$), 36.0 (*C*H$_2$), 35.4 (*C*H$_2$), 23.4 (*C*H$_2$), 21.6 (*C*H$_3$), 20.5 (*C*H$_3$); HRMS: *m/z* $[M + Na]^+$, found: 564.2156, calculated: 564.2164.

1.3 Chemoenzymatic preparation of 9

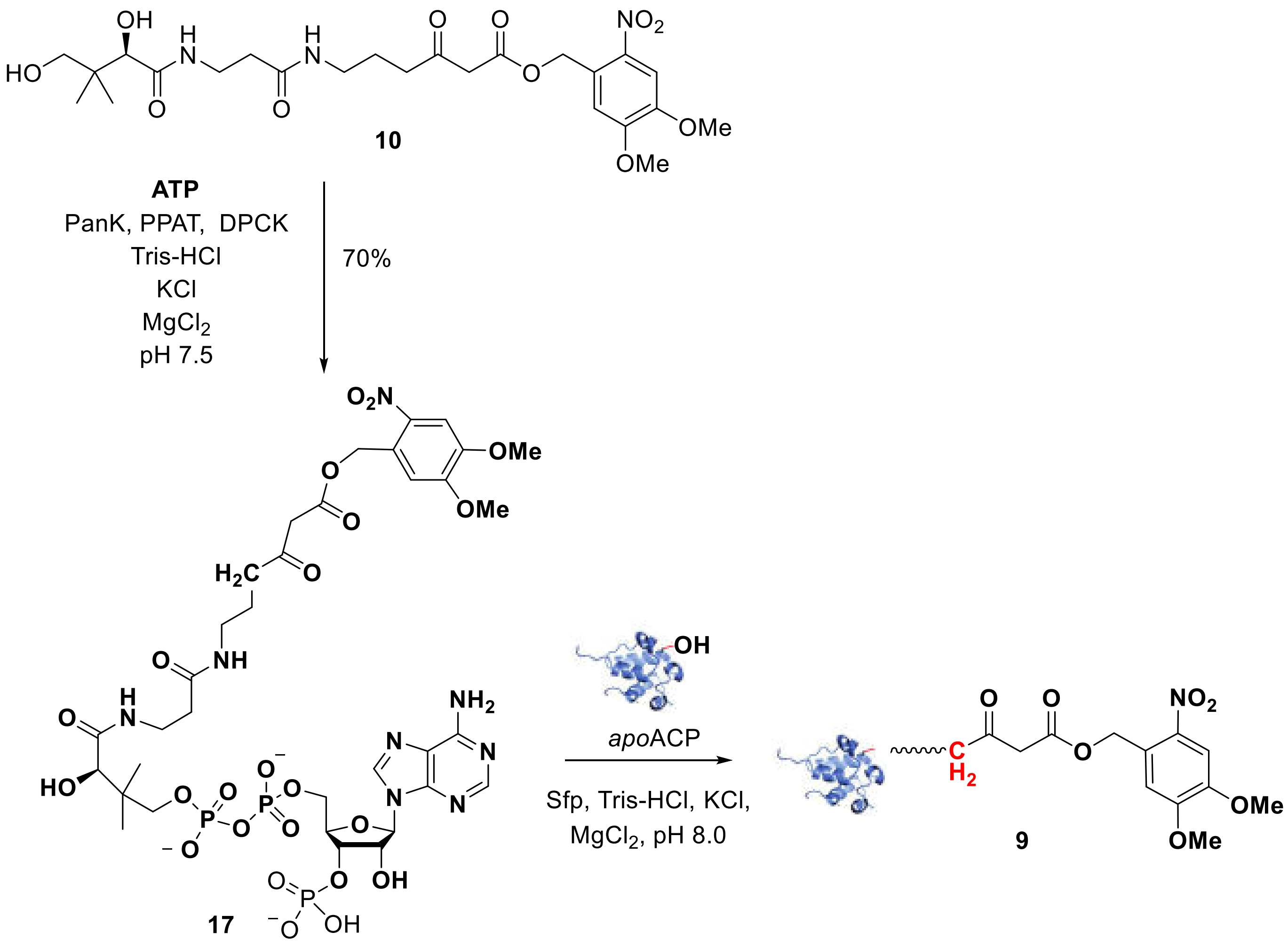

Scheme 2S: Preparation of **9.** Compound **10**, ATP, PanK, PPAT and DPCK enzymes [1] were incubated in a buffer containing Tris-HCl, KCl, $MgCl_2$, pH 7.5 at room temperature for 16 h and purified by semi-preparative HPLC to obtain **17**. Compound **17** and Sfp enzyme were incubated in a buffer containing Tris-HCl, KCl, $MgCl_2$, pH 8.0 at RT for 2 h to obtain the modified acyl carrier protein **9.**

PanK: pantothenate kinase

PPAT: phosphopantetheine adenylyltransferase

DPCK: dephosphocoenzyme A kinase

4,5-dimethoxy-2-nitrobenzyl 6-(3-((2*R*)-4-(((((5-(6-amino-9*H*-purin-9-yl)-4-hydroxy-3-(phosphonooxy)tetrahydrofuran-2-yl)

methoxy)(hydroxy)phosphoryl)oxy)(hydroxy)phosphoryl)oxy)-2-hydroxy-3,3-dimethylbutanamido)propanamido)-3-oxohexanoate (17)

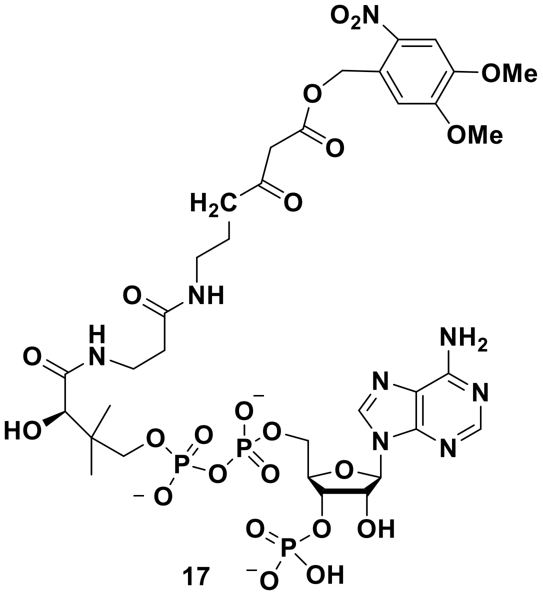

17

17 was prepared following a protocol for coenzyme A derivative preparation previously reported by us.[1] To a solution containing adenosine-5'-triphosphate (ATP) disodium salt (90 mg, 0.18 mmol), KCl (20 mM), $MgCl_2$ (10 mM) and compound **10** (15 mg, 0.28 mmol) in Tris-HCl (50 mM) at pH 7.5, the enzymes PanK, PPAT and DPCK (expressed and purified as indicated in section 3; final concentrations of 500 µg/mL, 500 µg/mL and 700 µg/mL respectively) were added to reach a final volume of 7.5 mL. After overnight incubation the enzymes were precipitated by addition of $CHCl_3$ (7.5 mL); the layers were separated and the aqueous phase was freezedried. The crude residue was purified by semi-preparative HPLC to yield **17** as a white powder (20 mg, 70% yield); R_t = 16.1 min.

^{1}H-NMR (500 MHz, D_2O) δ 8.57 (s, 1H, Ar*H*), 8.33 (s, 1H, Ar*H*), 7.64 (t, 1H, *J* 6.0 Hz, Ar*H*), 6.99 (t, 1H, *J* 5.0 Hz, Ar*H*), 6.08 (d, 1H, *J* 6.0 Hz, Ar*H*), 5.39 (s, 2H, C*H*$_2$), 4.78 (m, 1H, *J* 6.0 Hz, Ar*H*), 4.78 (m, 1H, Ar*H*), 4.51 (s, 1H, Ar*H*), 4.19 (s, 2H, C*H*$_2$), 3.96 (s, 1H, C*H*), 3.87 (s, 3H, OC*H*$_3$), 3.83 (s, 3H, OC*H*$_3$), 3.80 (m, 1H, *J* 9.5 Hz, C*H*$_2$), 3.53 (m, 1H, *J* 9.5 Hz, C*H*$_2$), 3.38 (t, 2H, *J* 6.5 Hz, C*H*$_2$), 3.04 (t, 2H, *J* 7.0 Hz, C*H*$_2$), 2.60 (t, 2H, *J* 7.0, 7.5 Hz, C*H*$_2$), 2.36 (t, 2H, *J* 6.5, 7.0 Hz, C*H*$_2$), 1.66 (q, 2H, *J* 7.0 Hz, C*H*$_2$), 0.87 (s, 3H, C*H*$_3$), 0.73 (s, 3H, C*H*$_3$); **^{13}C-NMR** (150 MHz, D_2O) δ 207.1 (*C*O), 174.7 (*C*ONH), 173.8 (*C*ONH), 168.9 (*C*OO), 153.1 (Ar*C*OCH$_3$), 149.8 (N*C*H), 148.3 (N*C*H), 147.6 (Ar*C*OCH$_3$), 144.4 (N*C*H), 142.3 (N*C*H), 139.6 (Ar*C*NO$_2$), 126.2 (Ar*C*), 118.4 (*C*NH$_2$), 111.1 (Ar*C*H), 108.4 (Ar*C*H), 87.5 (N*C*H), 83.5 (O*C*H), 74.1

(HO*C*H), 74.1 (PO*C*H), 74.1 (O*C*H), 71.9 (O*C*H_2), 71.9 (CO*C*H_2COO), 65.0 (O*C*H_2), 64.3 (Ar*C*H_2), 56.4 (O*C*H_3), 56.1 (O*C*H_3), 40.1 (*C*H_2), 38.4 (*C*H_2), 38.4 (*C*), 35.4 (*C*H_2), 35.3 (*C*H_2), 22.3 (*C*H_2), 20.8 (*C*H_3), 18.2 (*C*H_3); **^{31}P-NMR** (151 MHz, D_2O) δ 0.25 (1P, OPO_3H_2), -11.01 (2P, O(*P*$O_3H)_2$); **HRMS**: *m/z* $[M-2H]^{2-}$, found: 514.0974, calculated: 514.0978.

The photolabile coenzyme A derivative **17** was converted to the corresponding photolabile *act* ACP, tagged (Fig. 1S below) or untagged (**9**, Fig. 2S), as described in section 3.1.4. Hexahistidine-tagged photolabile ACP was subjected to purification by Ni affinity chromatography for MS characterisation (Fig. 1S): the crude enzymativ reaction was added to Ni-NTA spin columns (Thermo Scientific, HisPur Ni-NTA spin column, 0.2 mL resin bed), and left to mix on a rocker for 1 hour before the resin was washed 3 times with approximately 2 mL distilled water. The filtrate was then buffer exchanged with water to reduce salt concentration, and further concentrated using a centrifugal 3 kDa MWCO filter (Amicon) to the desired concentration for a stock solution. The samples were prepared immediately prior to FT-ICR-MS analysis by diluting in 50:50 acetonitrile: water with 0.1% formic acid to 2 µM concentration (direct infusion, as detailed in section 2).

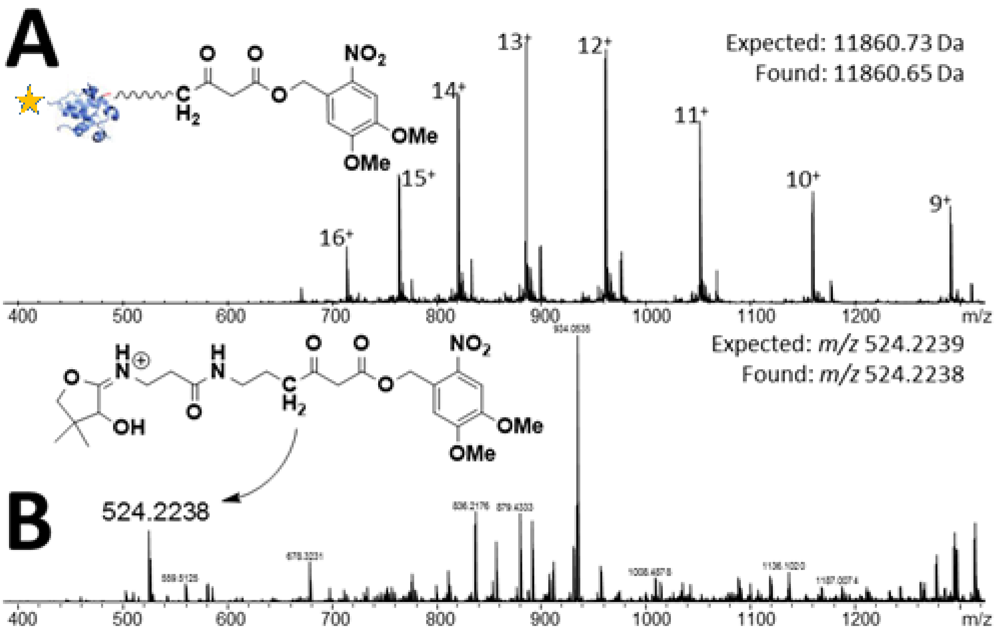

Figure 1S FT-ICR-MS characterisation of hexahistine-tagged photoactivatable *act*ACP: **A)** (Parent) mass spectrum, and **B)** CAD of the 12^{+} parent ion, generating a singly- charged pantetheine ion[2] containing the photolabile moiety.

2 Photolysis of 9 to generate carba(dethia) malonyl ACP (8)

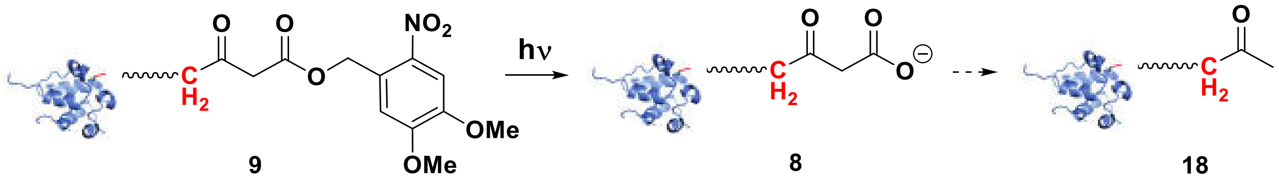

Scheme 3S

The photolysis of **9** (prepared as described above and in section 3.1.4) was successfully carried out following the procedures detailed below. Its outcome was estimated by monitoring the formation of **8** and its decarboxylation product **18** (Scheme 3S) by UPLC-HRMS (as detailed in section 3.1.4) and FT-ICR-MS analyses (below).

Photolysis of **9**: samples of **9** in 50 mM TrisCl, 20 mM KCl, 10 mM $MgCl_2$ pH 8 buffer with **9** at 40 µM were placed in a quartz cuvette for irradiation with an OBB Tunable KiloArc™ Illuminator at 365 nm and 1000W. Alternatively they were irradiated within a home-built light box equipped with a circular 22W UVA lamp (purposely built by Mr Rod Wesson, Electronics workshop, University of Warwick, UK). [3] Photolysis of **9** in the absence and in the presence of KS-CLF was carried out (Figures 2S and 3S). The samples were concentrated and buffer-exchanged using 3 kDa MWCO centrifugal filters with distilled water, then diluted to 2 µM with 50:50 acetonitrile:water (0.1% formic acid) ahead of HR-MS analyses.

FT-ICR-MS analysis: ultra-high resolution mass spectrometry data of untagged ACP species/ mixtures were recorded on a SolariX 12T FT-ICR mass spectrometer (Bruker Daltonik GmbH, Bremen, Germany), equipped with an Infinity cell. The FT-ICR MS data files were processed in absorption mode [4, 5] using previously described methods. [6, 7] The spectra were processed using an asymmetric apodization function (F = 0.4) to minimize baseline deviations in the resulting absorption mode spectrum but also allowing user control of the compromise between resolution and signal to noise during post-processing.[8] Peak picking was undertaken using AutoPiquer algorithm, with a user controlled S/N peak threshold of 1.5.[9, 10] Assignments were only made where all isotopologues that should be detectable, given the spectral resolution, the S/N of the most

abundant isotopologue and the peak picking threshold, were present in the peak list, within the user controlled mass error.

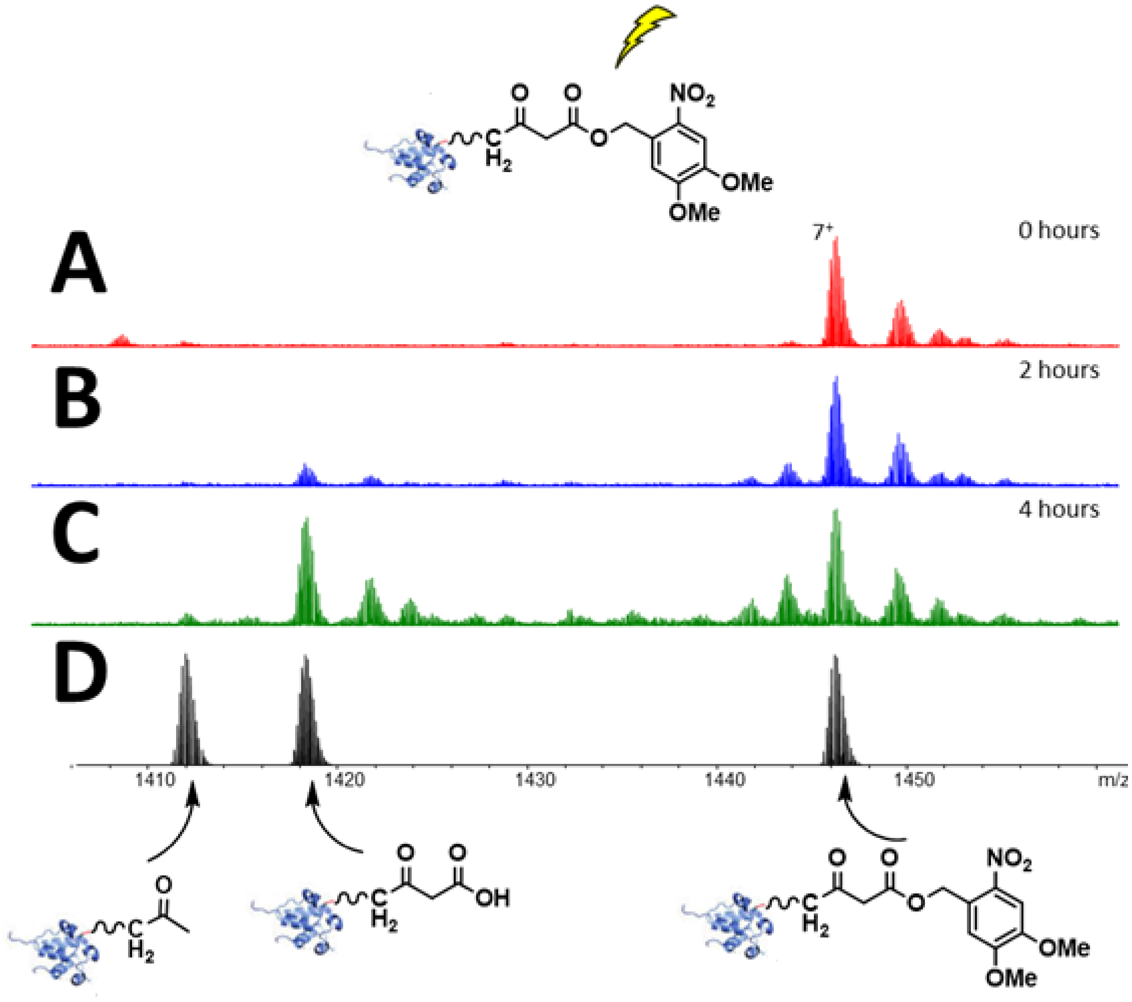

Figure 2S FT-ICR-MS analysis of **9** irradiated on its own (7^+ ions) at 365 nm, 1000 W, for 0 hours (**A**), 2 hours (**B**) and 4 hours (**C**) in 50:50 acetonitrile:water (0.1% formic acid) at 2 µM. (**D**) illustrates simulated isotope patterns for the 7^+ ions of the photolabile ACP **9**, carba(dethia) malonyl ACP **8** and its decarboxylated product **18**.

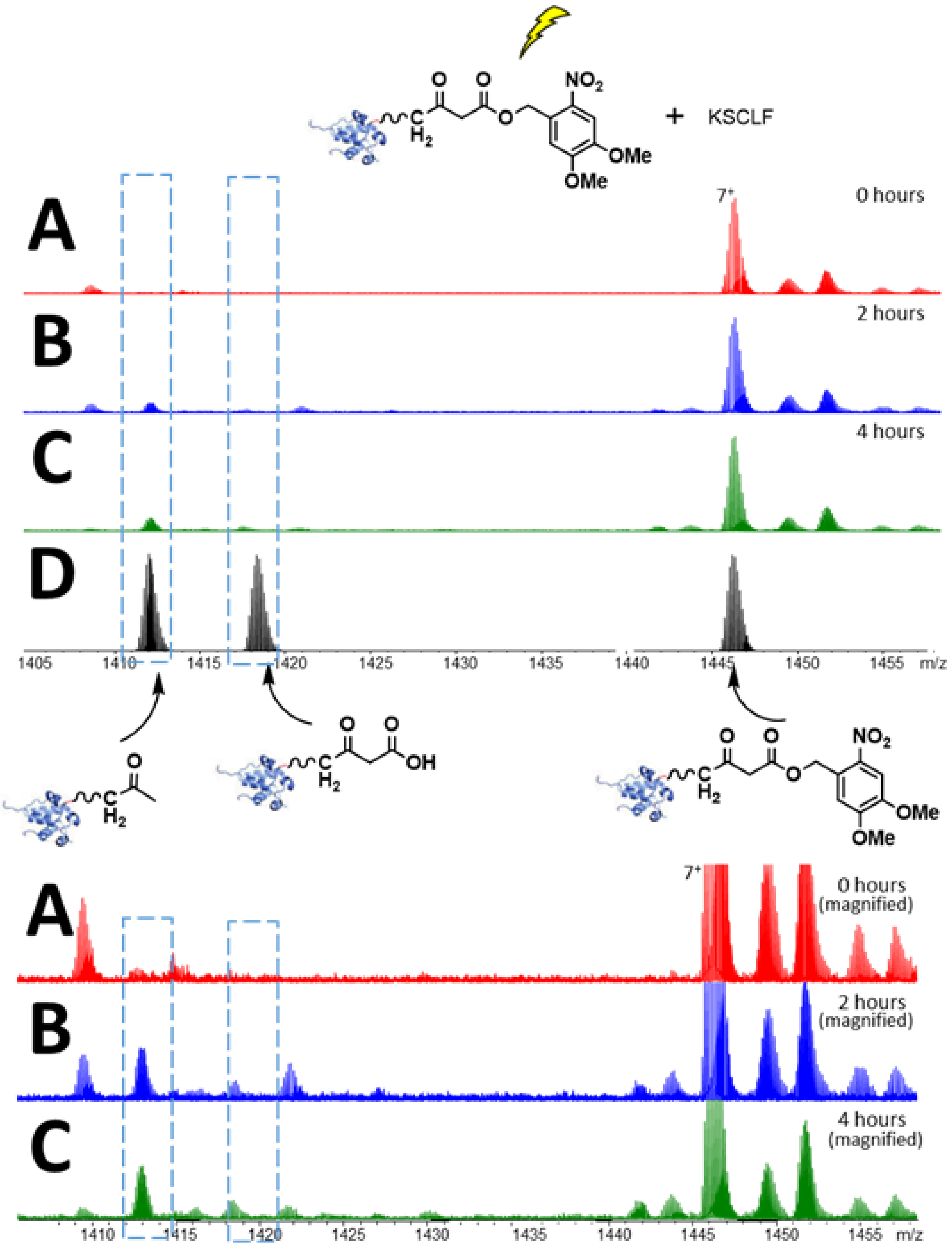

Figure 3S Top: FT-ICR-MS analysis of **9** (7^+ ions) irradiated at 365 nm, 1000 W, in the presence of KS-CLF for 0 hours (**A**), 2 hours (**B**) and 4 hours (**C**), in 50:50 acetonitrile:water (0.1% formic acid) at 2 µM. . (**D**) illustrates simulated isotope distributions for 7^+ ions.

Bottom: magnified **A-C** spectra showing the presence of carba(dethia) acetyl ACP **18** in low amount and the absence of carba(dethia)malonyl ACP **8**.

3 *In vitro* intermediate capture from the type II *act* minimal PKS *via* carba(dethia) malonyl ACP (8)

3.1 General methods

3.1.1 Expression and purification of hexahistidine-tagged proteins in *E. coli* (*act* apoACP, Sfp, PanK, PPAT and DPCK)

Hexahistidine-tagged proteins were expressed and purified from cultures of *E. coli* BL21*(DE3) strain transformed with plasmids pET28a-*act*ACP-C17S, pET28a-Sfp, pET28a-PanK, pET28a-PPAT, and pET29b-DPCK, and grown in autoclaved (Astell autoclave) LB broth media (Miller, BP1426-2, 25 g/L). Precultures (10 mL) of LB media containing the appropriate antibiotic (kanamycin, 25 µg/mL, for *act*ACP, Sfp, PanK and PPAT; carbenicillin, 100 µg/mL, for DPCK) were incubated overnight at 37 ^{o}C, at 180 rpm (Innova^{TM} 4300 Incubator Shaker, New Brunswick Scientific). The precultures were used to inoculate 1 L flasks of LB media with appropriate antibiotic (as previous), and incubated at 37 ^{o}C, 180 rpm (Innova$^{(R)}$ 44 Incubator shaker series) until an optical density at 600 nm between 0.5 and 0.8 was reached. At this point isopropyl β-D-1-thiogalactopyranoside (IPTG, 0.5 mM final concentration) was added to each flask to induce protein expression. The flasks were incubated at 15 ^{o}C and 180 rpm overnight (Innova$^{(R)}$ 44 Incubator shaker series). The cultures were centrifuged at 5000 rpm at 4 ^{o}C, for 15 minutes (Sorvall$^{(R)}$ RC6 Plus, SLA 3000 Super-lite rotor). The pellets, containing whole cells, were resuspended in binding buffer (100 mM KH_2PO_4, 20 mM imidazole, 10% glycerol, pH 7.3). The cells were lysed with a cell disruptor (Constant Systems, Ltd), at 20 kPSI, one shot. The lysed cells were then centrifuged at 20000 rpm for 30 minutes (Beckman Coulter Avanti^{TM} J-30I centrifuge). The supernatant was added charged Ni-sepharose^{TM} 6 Fast Flow resin (1 mL, equilibrated into the binding buffer), and mixed at 4 ^{o}C.

After 1 hour, the resin was washed with binding buffer (20 mL) to wash off loosely bound proteins, and finally elution buffer (6 mL, 100 mM KH_2PO_4, 500 mM imidazole, 10% glycerol, pH 7.3) was added to the column to elute the His-tagged proteins. This final fraction was buffer exchanged using a PD10 desalting column (GE Healthcare) into a storage buffer (50 mM Tris-HCl, 20 mM KCl, 10 mM $MgCl_2$, pH 8) and stored in 50% glycerol at -80 ^{o}C. SDS-PAGE analysis of the purified protein showed bands at approximately 11, 28, 39, 20 and 23 kDa as previously reported.[1, 4] Bradford assay

was used to determine protein concentration using bovine serum albumin (0.1 mg/mL to 0.7 mg/mL) as calibrant. Approximately 7 mg/L for Sfp and 5 mg/L for *act*ACP were obtained, as well as 7 mg/L, 52 mg/L, 2 mg/L respectively for PanK, PPAT and DPCK. [1]

3.1.2 Expression and purification of *act* KS-CLF in *S. coelicolor*

KS-CLF was expressed and purified from frozen *S. coelicolor* CH999 (previously transformed with KS-CLF expression vector pCB84, kind gift of Dr John Crosby, Bristol) grown in autoclaved (Astell autoclave) Super YEME media (1.500 g yeast extract, 2.500 g bacto peptone, 1.500 g malt extract, 5.000 g glucose, 170.000 g sucrose, 2.5 g glycine and 1.175 g $MgCl_2.6H_2O$ in 500 mL of deionised and autoclaved water, then 37.5 mg L-proline, 37.5 mg L-arginine, 37.5 mg L-cysteine, 50.0 mg L-histidine, 7.5 mg uracil were added). A preculture (10 mL) containing kanamycin (100 µg/ml) was incubated at 30 ^oC, 180 rpm for 5 days. This was subsequently used to inoculate a 250 mL super YEME containing kanamycin (100 µg/ml) and nalidixic acid (25 µg/ml), and incubated for a further 2 days at 30°C, 180 rpm. The culture was induced with thiostrepton in DMSO (10 µg/ml), and incubated further at 30 ^{o}C, 180 rpm for 5 days. The cells were pelleted at 9000 rpm for 25 mins, resuspended in binding buffer (100 mM KH_2PO_4, 10% glycerol, pH 7.3) and lysed with cell disruptor at 30 kPSI, one shot. The lysed cell suspension was centrifuged at 20000 rpm for 30 mins. Streptomycin sulphate (20 mg/ml) was added to the supernatant to precipitate the DNA, and mixed for 20 mins before centrifugation at 20000 rpm for 30 mins. The supernatant was purified by Ni-sepharose resin as described in paragraph 3.1.1. The buffers used were: wash buffer: 100 mM KH_2PO_4, 40 mM imidazole, 10% glycerol, pH 7.3; and elution buffer: 100 mM KH_2PO_4, 500 mM imidazole, 10% glycerol, pH 7.3. A PD10 column was used to buffer exchange the purified enzme into a storage buffer (100 mM KH_2PO_4, 2 mM DTT, 10% glycerol, pH 7.3), before storage in 50% glycerol at -80°C.

SDS-PAGE analysis of the purified protein showed two discrete bands at approximately 50 kDa and 43 kDa, as previously reported. [11] Bradford assay was used to determine protein concentration using bovine serum albumin (0.1 mg/mL to 0.7 mg/mL) as calibrant; the amount of protein obtained was estimated to be of approximately 45 mg/L.

3.1.3 Thrombin cleavage of hexahistidine-tag from *act* ACP

Hexahistidine *act*-apoACP (expressed and purified as above described) was subjected to tag cleavage *via* thrombin. For this a Thrombin CleanCleave Kit™ (Sigma), containing 50% slurry of immobilised bovine thrombin on agarose, was utilised. The resin was buffer-exchanged into cleavage buffer (10X: 500 mM Tris-HCl, pH 8.0, 100 mM $CaCl_2$) prior to use. 100 µL of thrombin-agarose slurry was employed to cleave 1 mg of target protein, at a concentration of 1 mg/mL (target protein) after six hours at room temperature with mixing by a see-saw rocker. The cleaved protein was purified from His-tagged protein by bench top purification with Ni-sepharose™ 6 Fast Flow resin, as described in 3.1.1. Cleavage of the His-tag was confirmed by SDS-PAGE and mass spectrometry.

3.1.4 Phosphopantetheinylation of act-*apo*ACPs

Unless otherwise stated, *apo*-ACP (tagged or untagged, 0.3 mM, final concentration), Sfp [12] (0.03 mM) and a coenzyme A derivative (malonyl CoA or the photolabile CoA derivative **17**, 0.7 mM) were incubated in Tris-HCl (50 mM), KCl (20 mM), $MgCl_2$ (10 mM), pH 8, at room temperature for 2 hours. Sample were monitored for complete phosphopantetheinylation by UPLC-MS analysis.

These were carried out on a Dionex UPLC system coupled to a Bruker MaXis mass spectrometer using the following materials/ conditions:

UPLC column: ACE C4-300 (UPLC)

Flow rate: 0.2 mL/min

Solvents (A/B) : H_2O/ACN containing 0.05% trifluoroacetic acid

Oven temperature: 30°C

Gradient: % B (ACN) varying as shown in table

Time (mins)	% B
0	5
5	5
10	40
40	45
50	100
55	100
60	5

3.1.5 Priming of *act* KS-CLF

Unless otherwise stated, KS-CLF (0.12 mM) was primed with acetyl-CoA (1.2 mM), *apo*-ACP (0.12 mM) and Sfp (0.012 mM) in Tris-HCl (50 mM) buffer, KCl (20 mM), $MgCl_2$ (10 mM), pH 8, at room temperature for 2 hours.

3.1.6 Reconstitution of enzymatic activity for the *act* PKS minimal system

Unless otherwise stated, the actinorhodin PKS minimal system was reconstituted *in vitro* as follows. Malonyl ACP **7** (6X His- tagged) and acetyl- KS-CLF (6X His- tagged) were generated immediately prior to this reaction. Malonyl ACP **7** (0.16 mM), acetyl-KSCLF (3 µM) and excess malonyl CoA (1 mM) were incubated in TCEP (1 mM), EDTA (2 mM) made to the desired volume (for the specified concentrations) with 100 mM phosphate buffer pH 7.3 at room temperature overnight. The pH was adjusted to approximately 4 adding concentrated HCl, and the aqueous layer extracted thrice with ethyl acetate. The ethyl acetate was removed *in vacuo* and the residue reconstituted in HPLC-grade acetonitrile/water for UPLC-MS analysis to assess the formation of SEK4 (**4**) and SEK4b (**5**). The extracts were analysed on a Dionex UPLC system coupled to a Bruker MaXis mass spectrometer (Eclipse plus C18, 1.8 µm, 2.1 x 100 mm UPLC column; flow rate: 0.2 mL; solvents: water, A, and acetonitrile, B, containing 0.1% formic acid; B gradient: 5% for 5 min, increased to 100% over 30 min, kept at 100% for 5 min, before equilibration to initial conditions over 10 min). The production of **4/5** was also verified in parallel monitoring their UV absorbance at 293 nm as previously reported (data not shown). Beside standard assays, the *act* PKS minimal system activity was also evaluated after subjecting the assays to irradiation at 365 nm: this did not affect the production of **4/ 5** (see below).

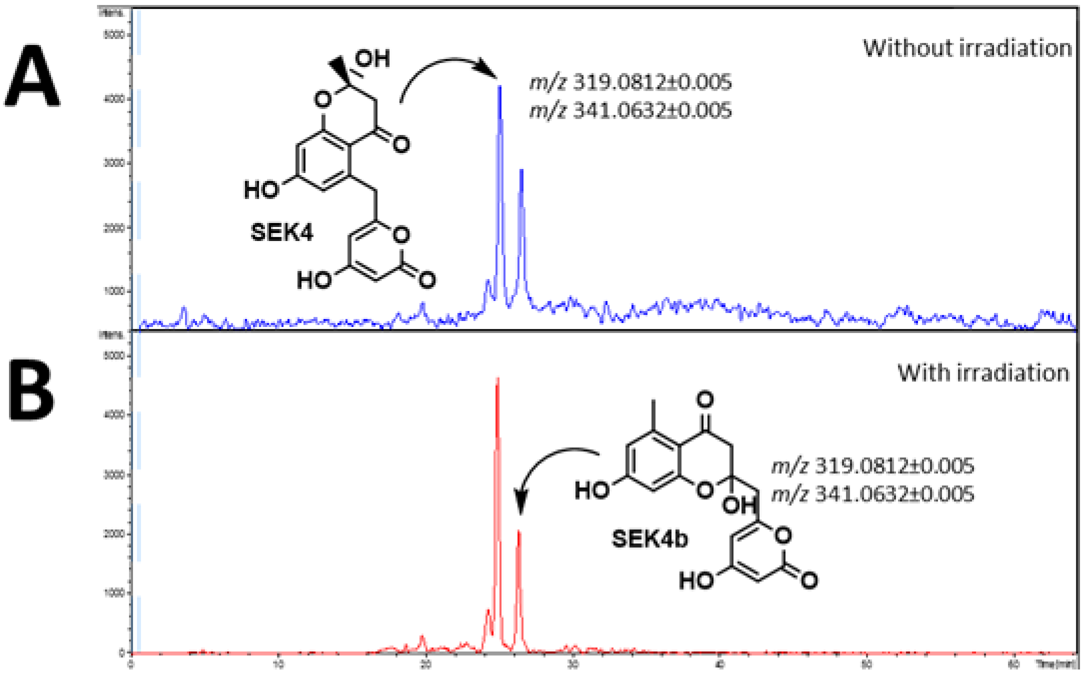

Figure 4S UPLC-MS chromatogram showing the protonated and sodiated EIC of the isomers SEK4 and SEK4b produced by the actinorhodin PKS minimal system. Peak assignments of SEK4 and SEK4b were based on previous literature.[11] **A**) Production of SEK4/4b (**4**/**5**) by the reconstituted actinorhodin PKS minimal system. **B**) Production of **4**/**5** by the same system subjected to photolysis for 2 hours at 365 nm, 1000W.

3.1.7 Intermediate capture from the *act* PKS minimal system *via* the ACP probe 8 and FT-ICR-MS characterization

Unless otherwise stated, actinorhodin PKS minimal system assays (comprising tagged malonyl ACP **7** and acetyl KS-CLF) were set up as described in section 3.1.6. Varying amounts of carba(dethia) malonyl ACP **8**, generated separately by photolysis of **9** as described in section 2, were present in the assays either from the very beginning or added afterwards (delayed addition). In some cases, $^{13}C_3$-labelled malonyl ACP **19** (below) alone was utilised with KS-CLF to generate labelled products.

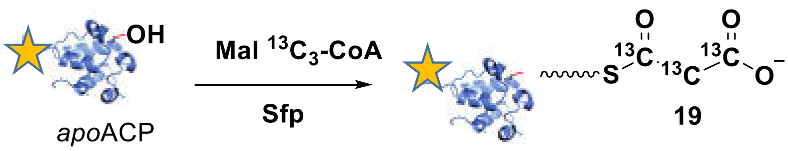

An overview of all experiments set up for intermediate capture from the type II *act* PKS minimal system is provided in Table 1S.

Table 1S: overview of putative biosynthetic intermediate capture experiments in the assembly of **4/5** *via* carba(dethia) malonyl ACP **8** (generated by photolysis of **9**).

	probe **9**[a] : malonyl ACP **7**					probe **9**[a] : $^{13}C_3$ labelled malonyl ACP **19**[d]			
**Putative ACP-bound intermediates detected**	1:1	1:4 step-wise [b]	5:1	1:1 30s delay[c]	1:1 5m delay[c]	1:1	1:10	5:1	1:4 step-wise[b]
**Diketide**									
Triketide									
**Tetraketide**									
**Pentaketide**									
**Hexaketide**									
Heptaketide									
Octaketide									
Dehydro tetraketide									
Didehydro tetraketide									
**Dehydro pentaketide**									
**Didehydro (cyclised) pentaketide**									
**Dehydro hexaketide**									
**Didehydro (cyclised) hexaketide**									
Dehydro heptaketide									
Didehydro heptaketide									
Dehydro octaketide									
Didehydro octaketide									
Tridehydro octaketide									

[a] photolysed to **8** over 4 hours on its own, then minimal PKS assay components added; [b] "Step-wise" indicated the reverse and gradual addition of the actinorhodin minimal system enzymes to an irradiating added hourly until 4 hours and 4 eq of the actinorhodin minimal system had been added to 1 eq of **8**. [c] 30s and 5m delay refer to the time delay upon addition of **9** to the actinorhodin minimal system (to allow the minimal system to react before adding the probe **9**). [d] used in assays on its own with KS-CLF.

Parallel control experiments (for which either minimal PKS assay components were missing/inactive, or **9** was unphotolysed) were also carried out. The assays were worked up *via* the use of Ni-NTA spin columns (Thermo Scientific, HisPur Ni-NTA spin column, 0.2 mL resin bed): the enzymatic samples were incubated with the Ni-NTA resin for 1 hour at 4°C, after which the resin was washed thrice with distilled H_2O (while spinning in a microcentrifuge) to elute any species derived from **8**. All other His-tagged proteins remained bound to the Ni resin. The filtrates were

buffer-exchanged with 3 kDa MWCO filters into water, and concentrated to provide a stock solution. Immediately prior to analysis, sample solutions were prepared at 2 µM in 50:50 acetonitrile: water, 0.1% formic acid for direct injection into an FT-ICR-MS instrument details as described in section 2. Where the putative detected intermediates trapped by the ACP probe **8** were of very low intensity, additional data analysis of the isotopic distributions was performed to confirm their presence as 'real' peaks by performing autocorrelation of the region in the spectrum.

Figure 1 presents an example of the autocorrelation of a very intense region of the spectrum, the 8^+ isotopic distribution of the signal for the ACP probe **8**. A large peak in the autocorrelation spectrum (inset) can be seen at a charge state of 8. For comparison, a region of known 'noise' in the spectrum, at high *m/z*, was chosen and, as expected, no autocorrelation can be seen for that region (Figure 2).

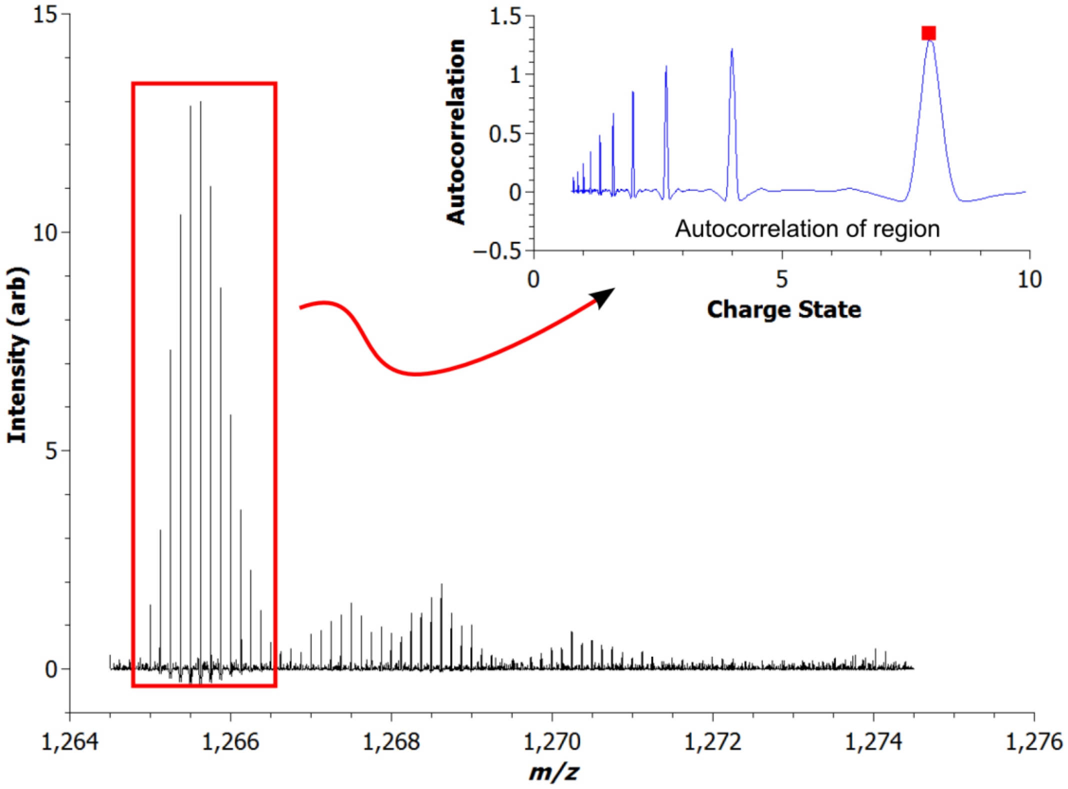

Figure 1 Example of a region of a spectrum with a very intense peak (ACP probe 8 at charge state 8^+) with the inset showing the auto-correlation of the region indicated, confirming the 8^+ charge state.

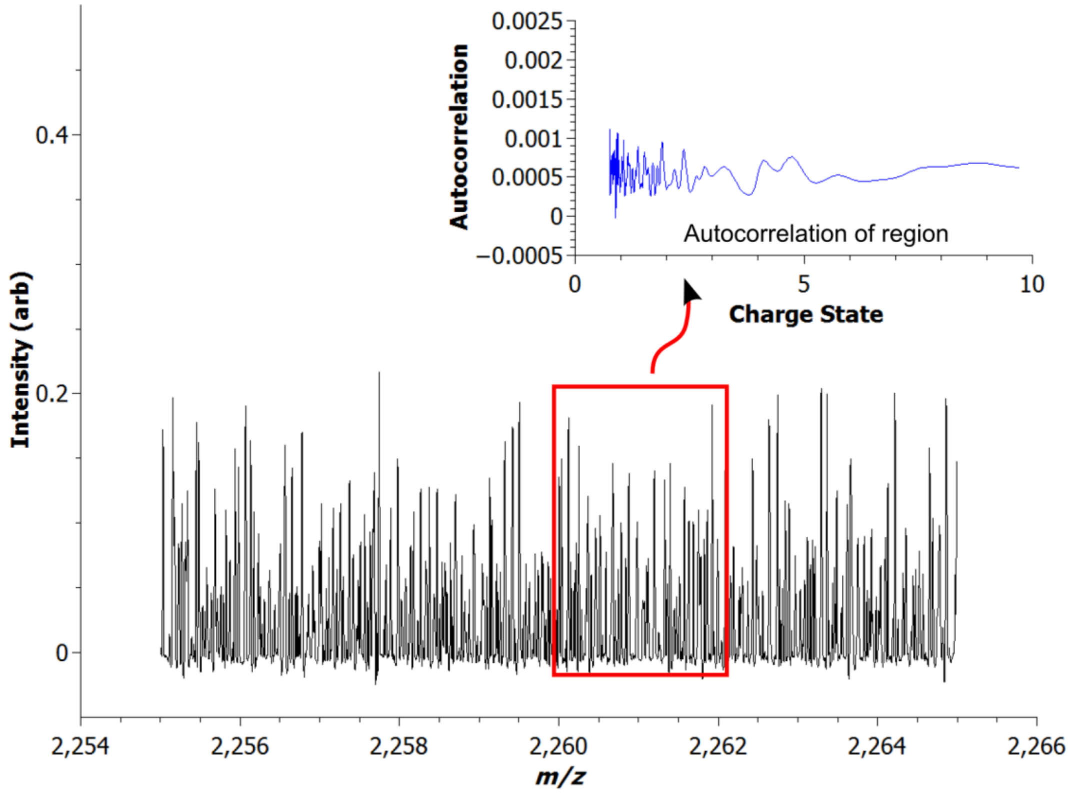

Figure 2 A blank region of a spectrum with the inset showing the auto-correlation of the region indicated, confirming that no isotopic distributions were detected, and this region of the spectrum is, indeed, noise.

The autocorrelation algorithm was applied to those regions of low intensity that had been previously manually identified to contain isotopic distributions for putative captured intermediates by the ACP probe **8** (selected figures to follow).

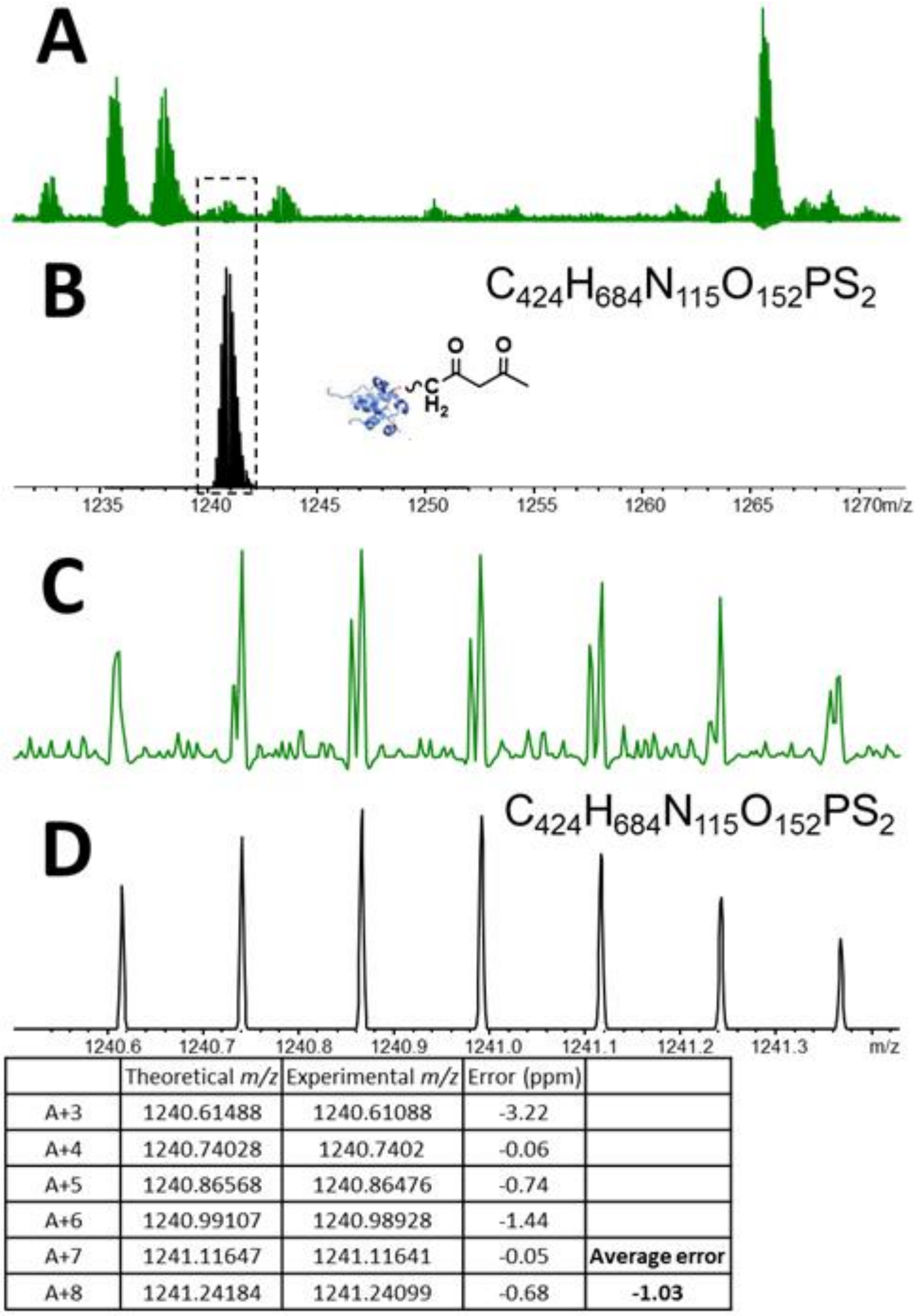

	Theoretical *m/z*	Experimental *m/z*	Error (ppm)	
A+3	1240.61488	1240.61088	-3.22	
A+4	1240.74028	1240.7402	-0.06	
A+5	1240.86568	1240.86476	-0.74	
A+6	1240.99107	1240.98928	-1.44	
A+7	1241.11647	1241.11641	-0.05	**Average error**
A+8	1241.24184	1241.24099	-0.68	**-1.03**

Figure 7S FTICR-MS analyses of carba(dethia)malonyl ACP **8** incubated in actinorhodin minimal system enzymatic assays (in 1: 1 ratio with malonyl ACP **7**) revealed the presence of a putative ACP-bound captured diketide: (**A**) acquired spectrum, (**B**) simulated spectrum, (**C**) magnified 8^+ ion and (**D**) its simulated isotope distribution with peak list and errors.

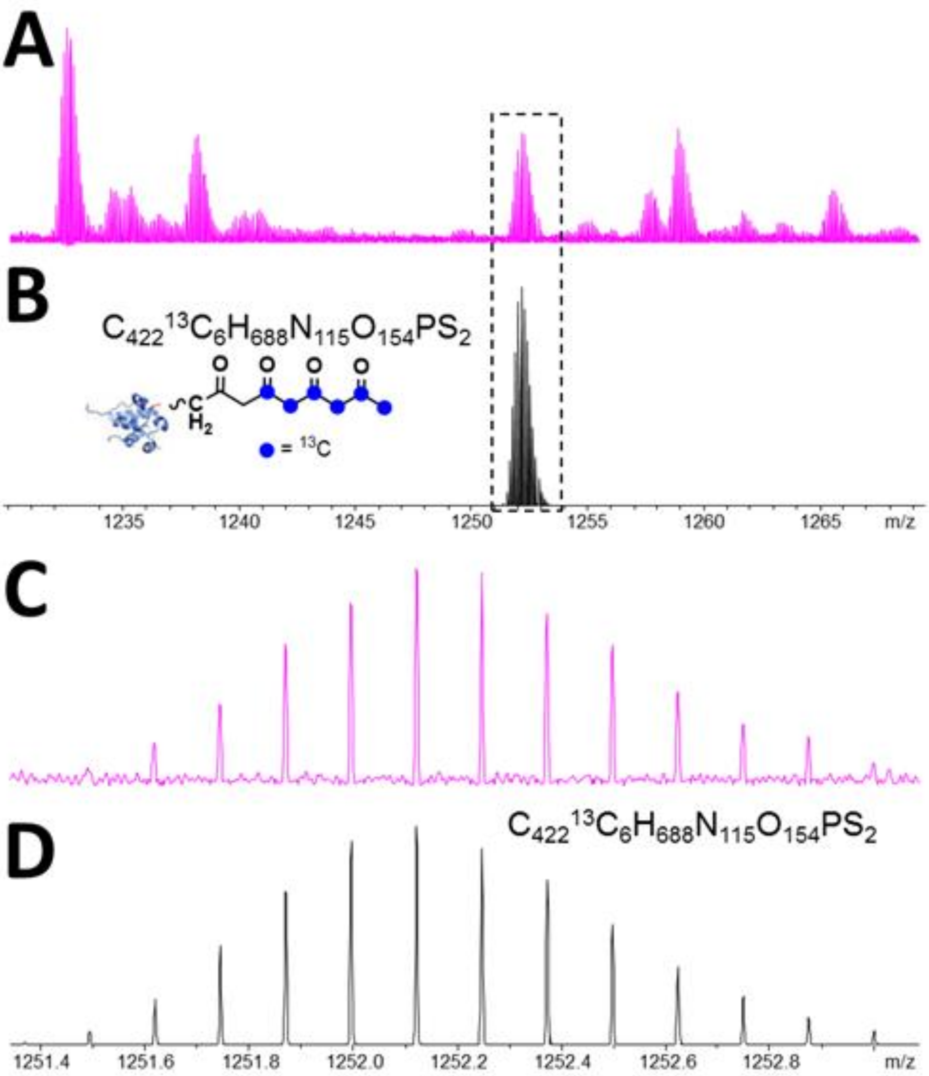

	Theoretical *m/z*	Experimental *m/z*	Error (ppm)	
A+3	1251.74462	1251.74349	-0.90	
A+4	1251.87003	1251.86902	-0.81	
A+5	1251.99543	1251.9947	-0.58	
A+6	1252.12083	1252.12025	-0.46	
A+7	1252.24622	1252.24565	-0.46	**Average error**
A+8	1252.37162	1252.37089	-0.58	**-0.63**

Figure 8S FTICR-MS analysis of carba(dethia)malonyl ACP **8** incubated in the actinorhodin minimal enzymatic assays (in 1:10 ratio with $^{13}C_3$-labelled malonyl ACP **19**) revealed the presence of a putative ACP-bound labelled tetraketide. (**A**) Acquired spectrum, (**B**) simulated spectrum, (**C**) magnified 8$^+$ ion and (**D**) its simulated isotope distribution with peak list and errors. Additional spectra and data for 6$^+$, 9$^+$ and 10$^+$ ions available on request.

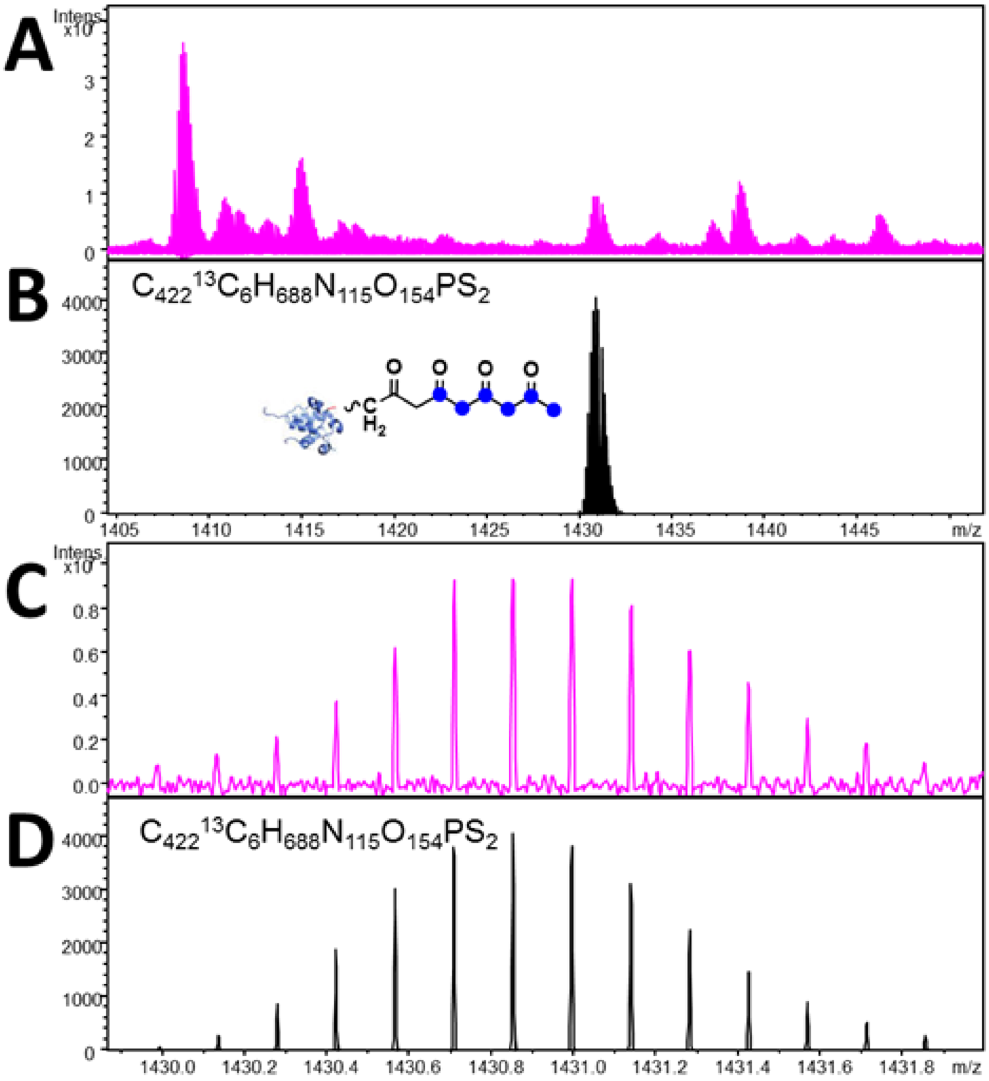

	Theoretical *m/z*	Experimental *m/z*	Error (ppm)	
A+3	1430.42138	1430.42117	-0.15	
A+4	1430.56471	1430.56471	0.00	
A+5	1430.70803	1430.70824	0.15	
A+6	1430.85134	1430.85093	-0.29	
A+7	1430.99464	1430.99435	-0.20	**Average error**
A+8	1431.13796	1431.13797	0.01	**-0.08**

Figure 9S FTICR-MS analysis of carba(dethia)malonyl ACP **8** incubated in the actinorhodin minimal enzymatic assays (in 1:10 ratio with $^{13}C_3$-labelled malonyl ACP **19**) revealed the presence of a putative ACP-bound labelled tetraketide. (**A**) Acquired spectrum, (**B**) simulated spectrum, (**C**) magnified 7^+ ion and (**D**) its simulated isotope distribution with peak list and errors.

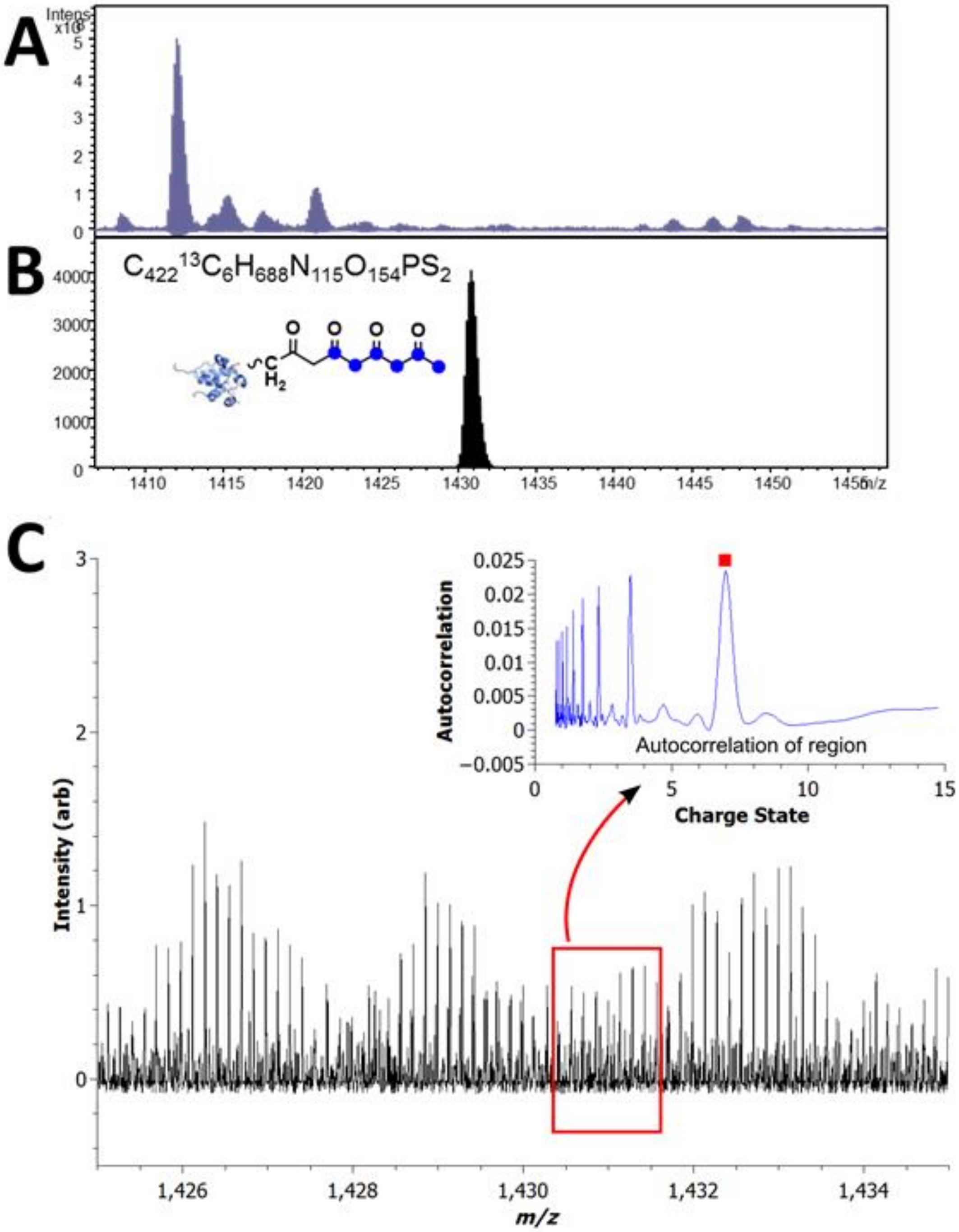

Figure 10S FTICR-MS analysis of carba(dethia)malonyl ACP **8** incubated in the actinorhodin minimal enzymatic assays (in 1:1 ratio with $^{13}C_3$-labelled malonyl ACP **19**) revealed the presence of a putative ACP-bound labelled tetraketide. (**A**) Acquired spectrum, (**B**) simulated spectrum and (**C**) autocorrelation of region confirming a 7+ charge state.

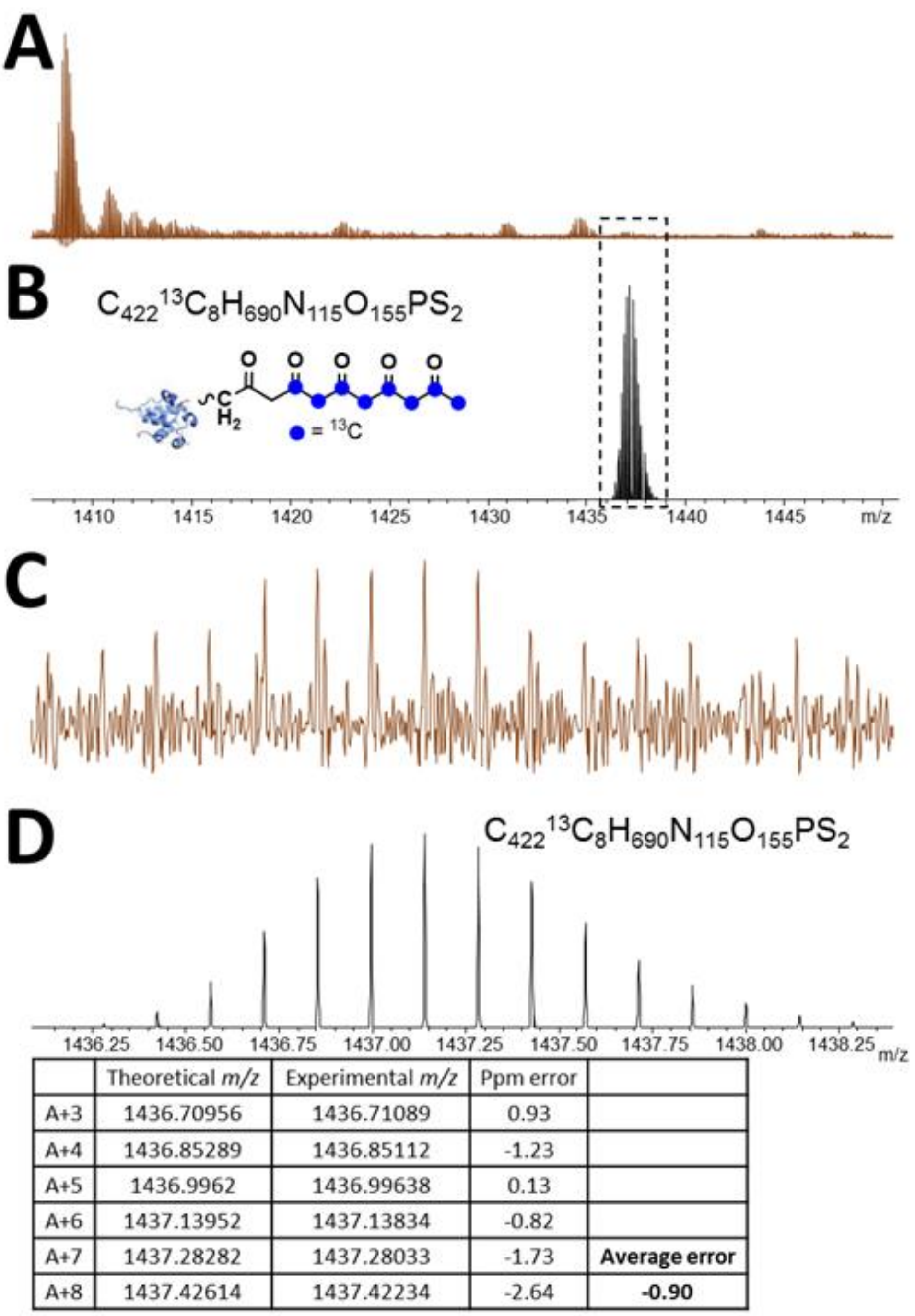

	Theoretical *m/z*	Experimental *m/z*	Ppm error	
A+3	1436.70956	1436.71089	0.93	
A+4	1436.85289	1436.85112	-1.23	
A+5	1436.9962	1436.99638	0.13	
A+6	1437.13952	1437.13834	-0.82	
A+7	1437.28282	1437.28033	-1.73	**Average error**
A+8	1437.42614	1437.42234	-2.64	**-0.90**

Figure 11S FTICR-MS analyses of carba(dethia)malonyl ACP **8** incubated in actinorhodin minimal system enzymatic assays (in 1: 4 ratio with ^{13}C-labelled malonyl ACP **19**, added stepwise) revealed the presence of a putative ^{13}C-labelled ACP-bound captured pentaketide: (**A**) acquired spectrum, (**B**) simulated spectrum, (**C**) magnified 7^+ ion and (**D**) its simulated isotope distribution with peak list and errors.

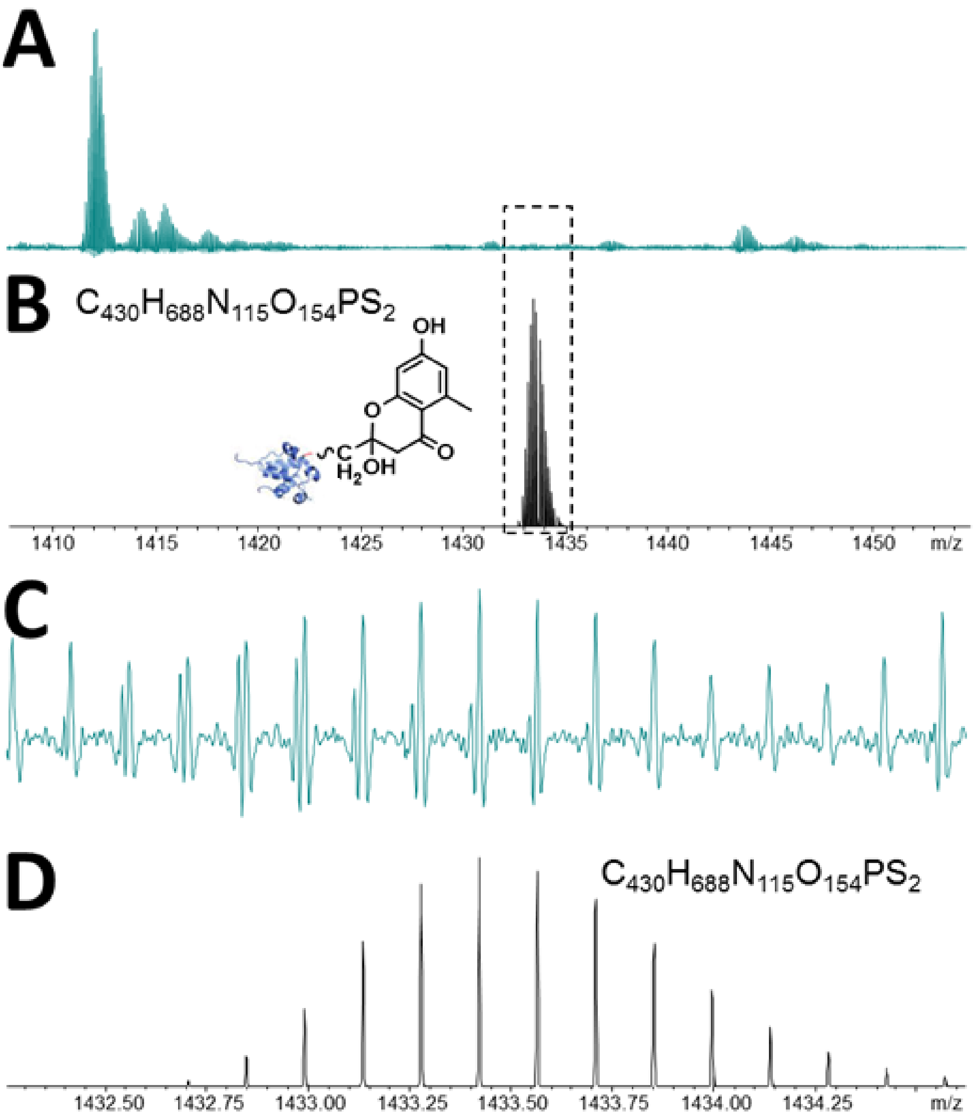

	Theoretical *m/z*	Experimental *m/z*	Error (ppm)	
A+3	1433.13327	1433.13271	-0.39	
A+4	1433.27659	1433.27623	-0.25	
A+5	1433.4199	1433.41998	0.06	
A+6	1433.56321	1433.56222	-0.69	
A+7	1433.70652	1433.70564	-0.61	**Average error**
A+8	1433.8498	1433.84896	-0.59	**-0.41**

Figure 12S FTICR-MS analyses of carba(dethia)malonyl ACP **8** incubated in actinorhodin minimal system enzymatic assays (in 5: 1 ratio with malonyl ACP **7**) revealed the presence of a putative ACP-bound captured dehydro (possibly cyclised) pentaketide: (**A**) acquired spectrum, (**B**) simulated spectrum, (**C**) magnified 7^+ ion and (**D**) its simulated isotope distribution with peak list and errors. Additional spectra and data for 6^+ and 8^+ ions available on request.

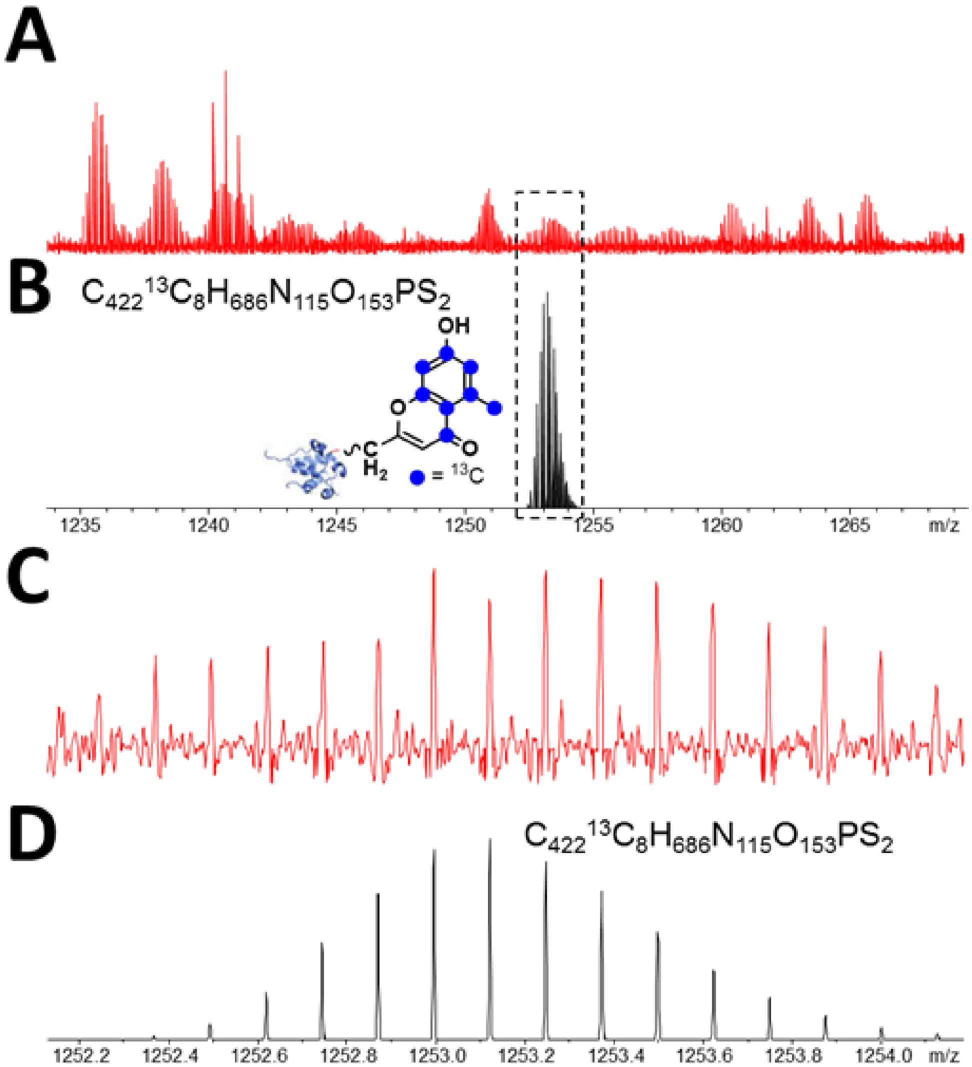

	Theoretical *m/z*	Experimental *m/z*	Error (ppm)	
A+3	1252.74413	1252.74834	3.36	
A+4	1252.86954	1252.87019	0.52	
A+5	1252.99495	1252.9946	-0.28	
A+6	1253.12015	1253.11904	-0.89	
A+7	1253.24574	1253.24477	-0.77	**Average error**
A+8	1253.37114	1253.36798	-2.52	**-0.10**

Figure 13S FTICR-MS analyses of carba(dethia)malonyl ACP **8** incubated in actinorhodin minimal system enzymatic assays (in 5: 1 ratio with ^{13}C-labelled malonyl ACP **19**) revealed the presence of a putative ACP-bound possibly cyclised didehydro pentaketide: (**A**) acquired spectrum, (**B**) simulated spectrum, (**C**) magnified 8^+ ion and (**D**) its simulated isotope distribution with peak list and errors. Additional spectra and data for 6^+, 7^+ and 10^+ ions available on request.

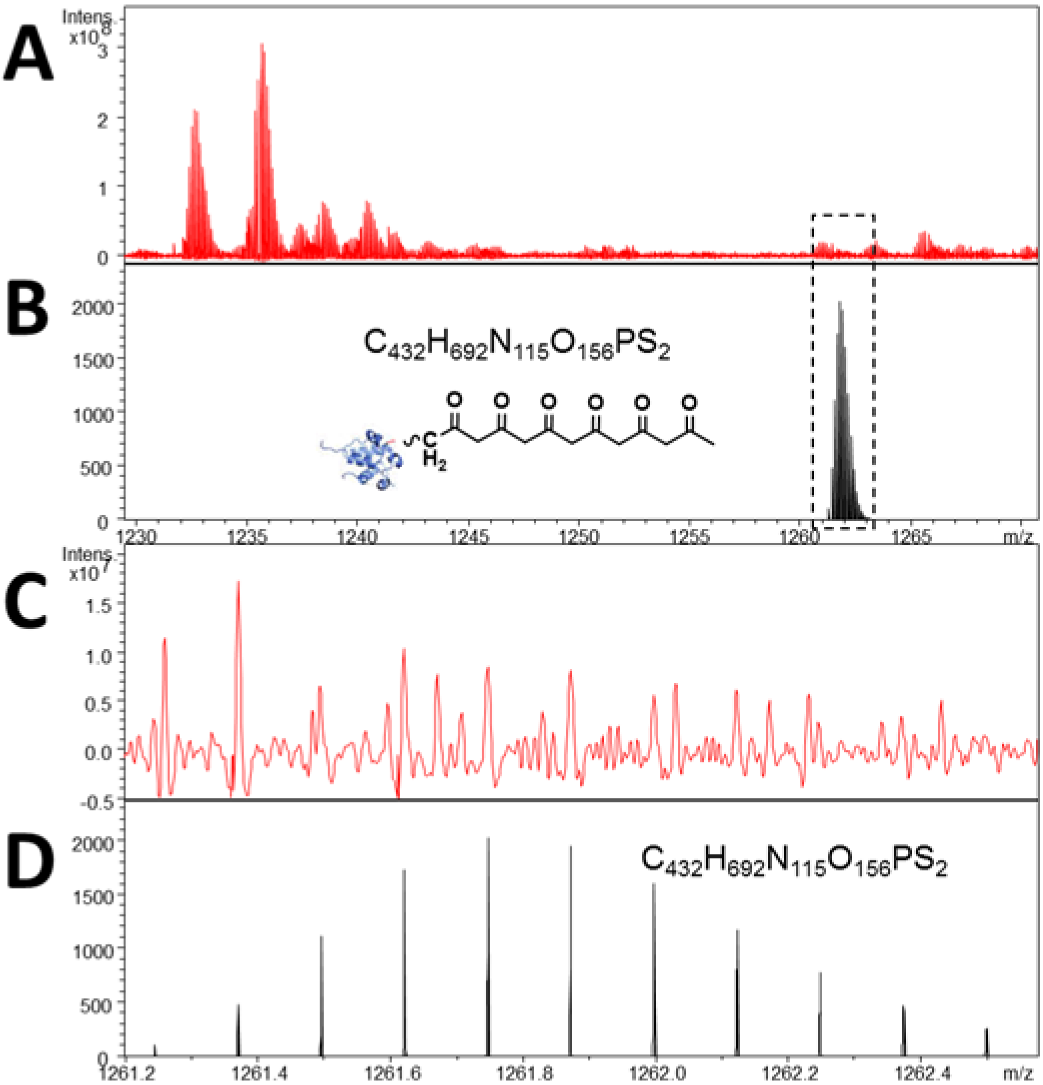

	Theoretical *m/z*	Experimental *m/z*	Error (ppm)	
A+3	1261.62016	1261.61952	-0.51	
A+4	1261.74558	1261.74539	-0.15	
A+5	1261.87096	1261.87056	-0.32	
A+6	1261.99636	1261.99651	0.12	
A+7	1262.12175	1262.1204	-1.07	**Average error**
A+8	1262.24712	1262.24384	-2.60	**-0.75**

Figure 14S FTICR-MS analyses of carba(dethia)malonyl ACP **8** incubated in actinorhodin minimal system enzymatic assays (in 1: 4 ratio with malonyl ACP **7**, added stepwise) revealed the presence of a putative ACP-bound captured hexaketide: (**A**) acquired spectrum, (**B**) simulated spectrum, (**C**) magnified 8^+ ion and (**D**) its simulated isotope distribution with peak list and errors.

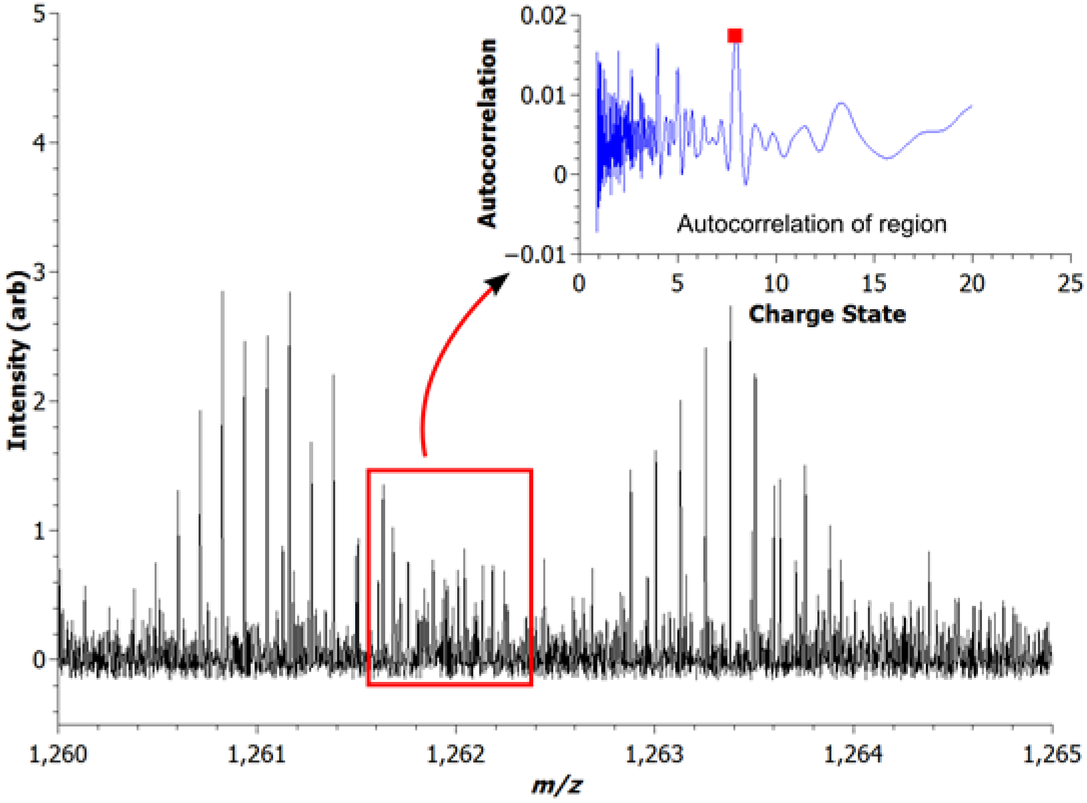

Figure 15S Autocorrelation analysis confirming an 8^{+} charge state for the putative ACP-bound hexaketide of Fig. 12S. Additional spectra, data and correlation analyses for 6^{+} and 7^{+} ions available on request.

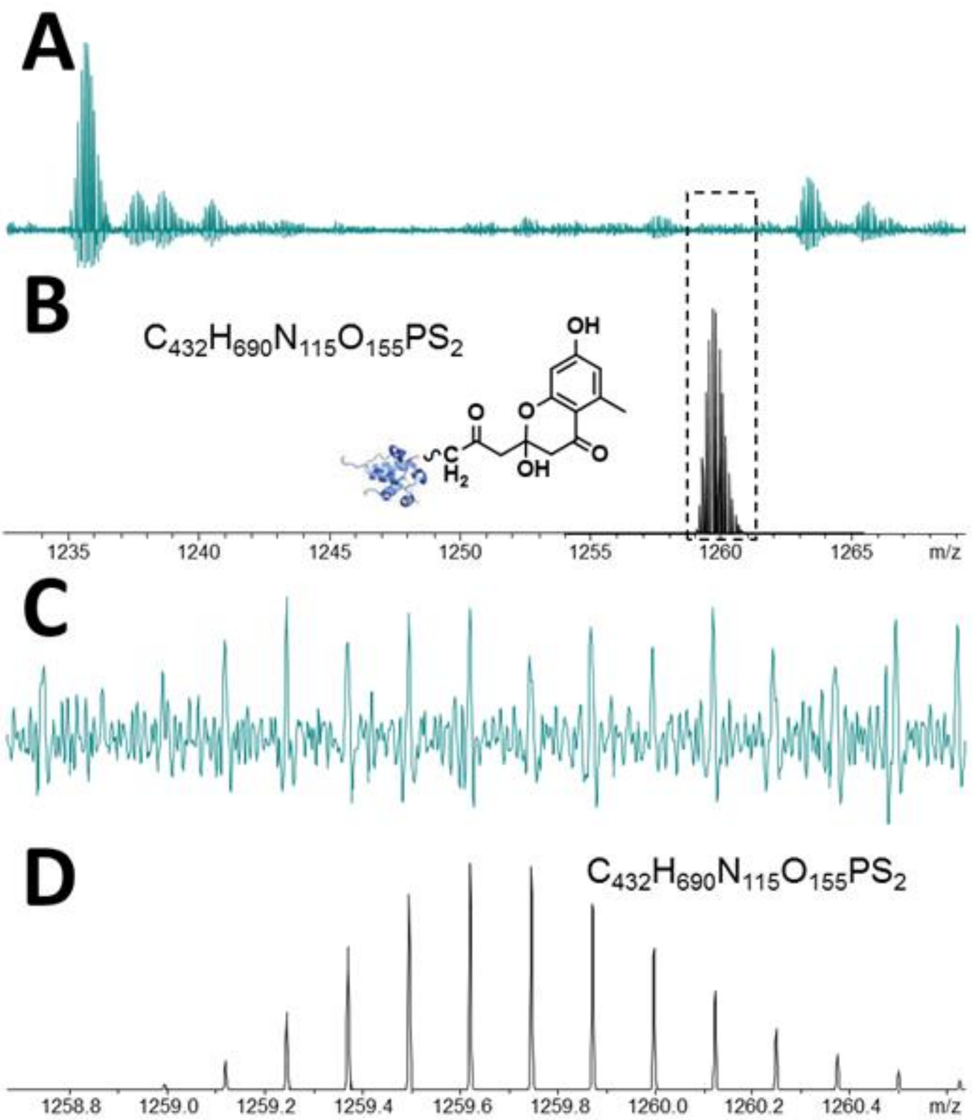

	Theoretical *m/z*	Experimental *m/z*	Error (ppm)	
A+3	1259.36884	1259.3678	-0.83	
A+4	1259.49424	1259.4935	-0.59	
A+5	1259.61964	1259.61923	-0.33	
A+6	1259.74504	1259.74627	0.98	
A+7	1259.87043	1259.86563	-3.81	**Average error**
A+8	1259.9958	1259.99272	-2.44	**-0.19**

Figure 16S FTICR-MS analyses of carba(dethia)malonyl ACP **8** incubated in actinorhodin minimal system enzymatic assays (in 5: 1 ratio with malonyl ACP **7**) revealed the presence of a putative ACP-bound captured dehydro (possibly cyclised) hexaketide: (**A**) acquired spectrum, (**B**) simulated spectrum, (**C**) magnified 8^+ ion and (**D**) its simulated isotope distribution with peak list and errors.

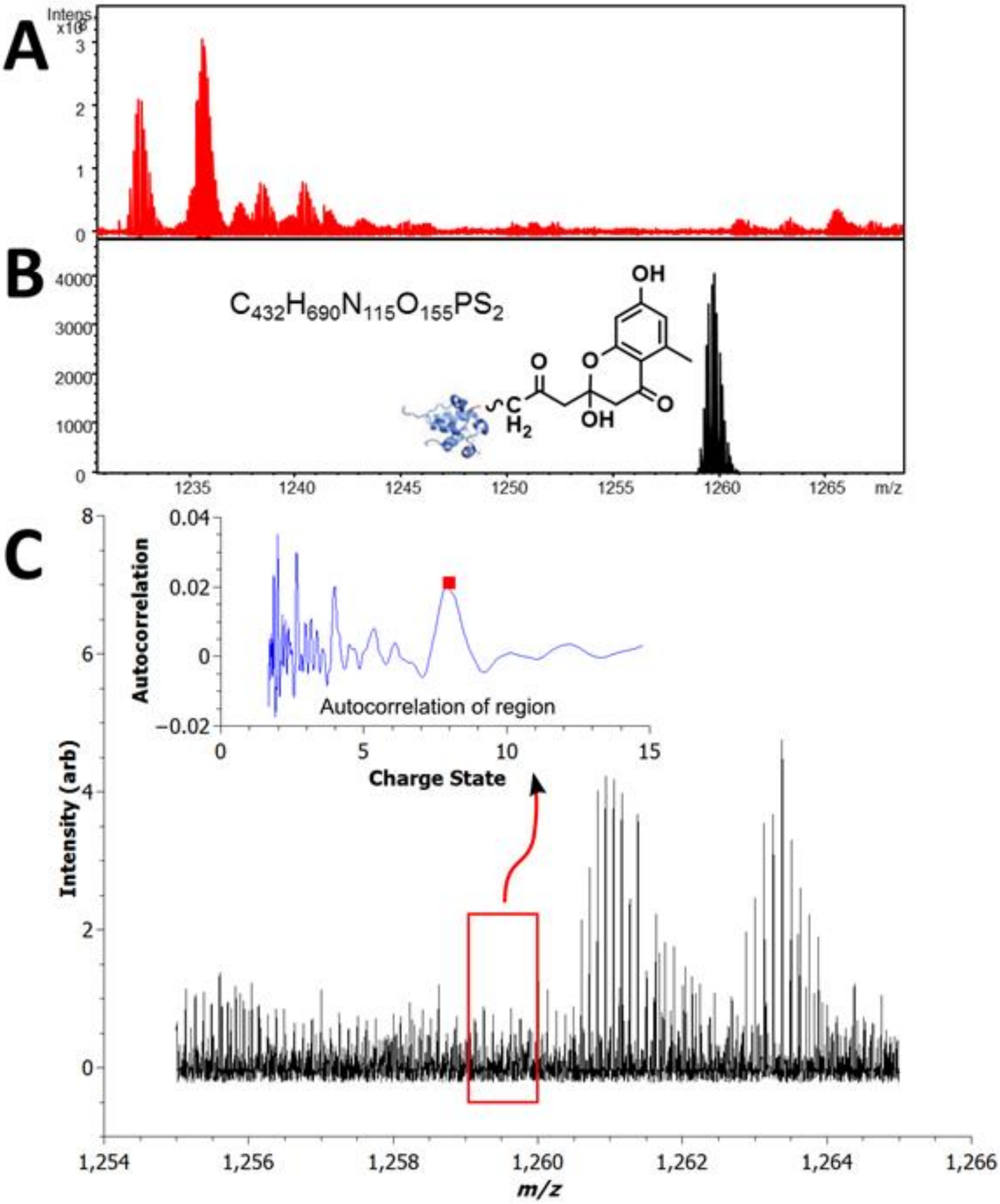

Figure 17S FTICR-MS analyses of carba(dethia)malonyl ACP **8** incubated in actinorhodin minimal system enzymatic assays (in 1: 4 ratio with malonyl ACP **7**, added stepwise) revealed the presence of a putative ACP-bound captured dehydro (possibly cyclised) hexaketide: (**A**) acquired spectrum, (**B**) simulated spectrum, and (**C**) autocorrelation of region confirming a 8^+ charge state. Additional spectra, data and autocorrelation analyses for 7^+ and 8^+ charge states available on request.

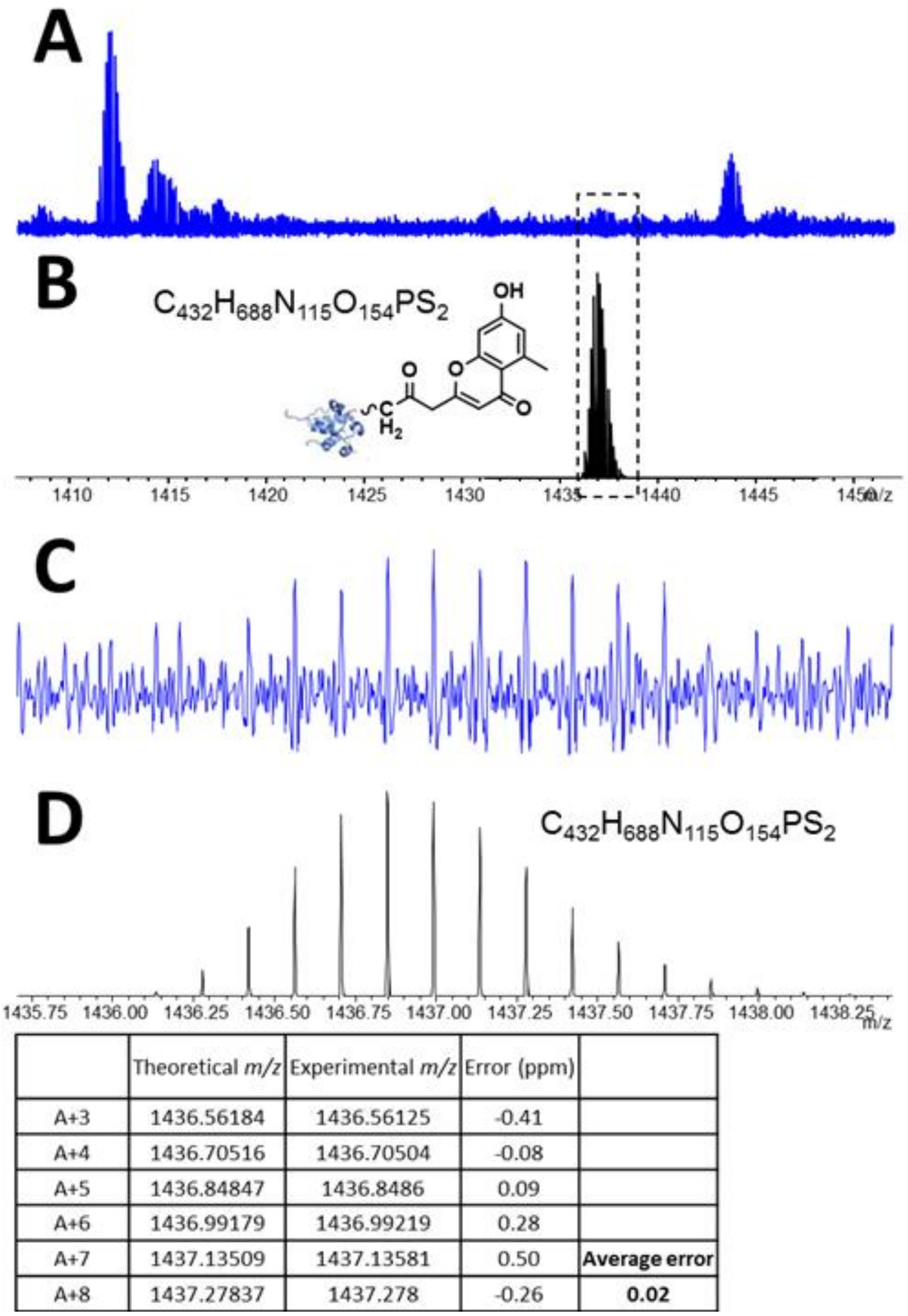

	Theoretical *m/z*	Experimental *m/z*	Error (ppm)	
A+3	1436.56184	1436.56125	-0.41	
A+4	1436.70516	1436.70504	-0.08	
A+5	1436.84847	1436.8486	0.09	
A+6	1436.99179	1436.99219	0.28	
A+7	1437.13509	1437.13581	0.50	**Average error**
A+8	1437.27837	1437.278	-0.26	**0.02**

Figure 18S FTICR-MS analyses of carba(dethia)malonyl ACP **8** incubated in actinorhodin minimal system enzymatic assays (in 1: 1 ratio with malonyl ACP **7**) revealed the presence of a putative ACP-bound captured didehydro (possibly cyclised) hexaketide: (**A**) acquired spectrum, (**B**) simulated spectrum, (**C**) magnified 7^+ ion and (**D**) its simulated isotope distribution with peak list and errors. Additional spectra, data and autocorrelation analyses for 6^+, 7^+ and 8^+ charge states available on request.

4 NMR spectra

4.1 ^{1}H- and ^{13}C- NMRs of compound 14

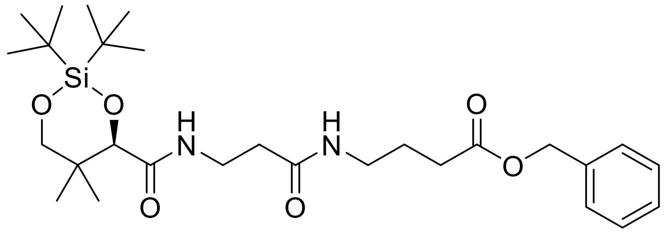

14

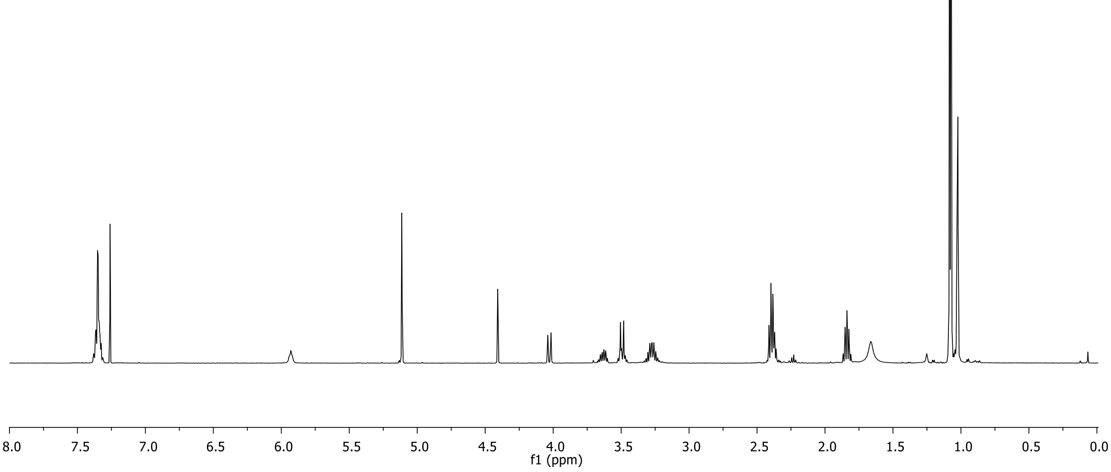

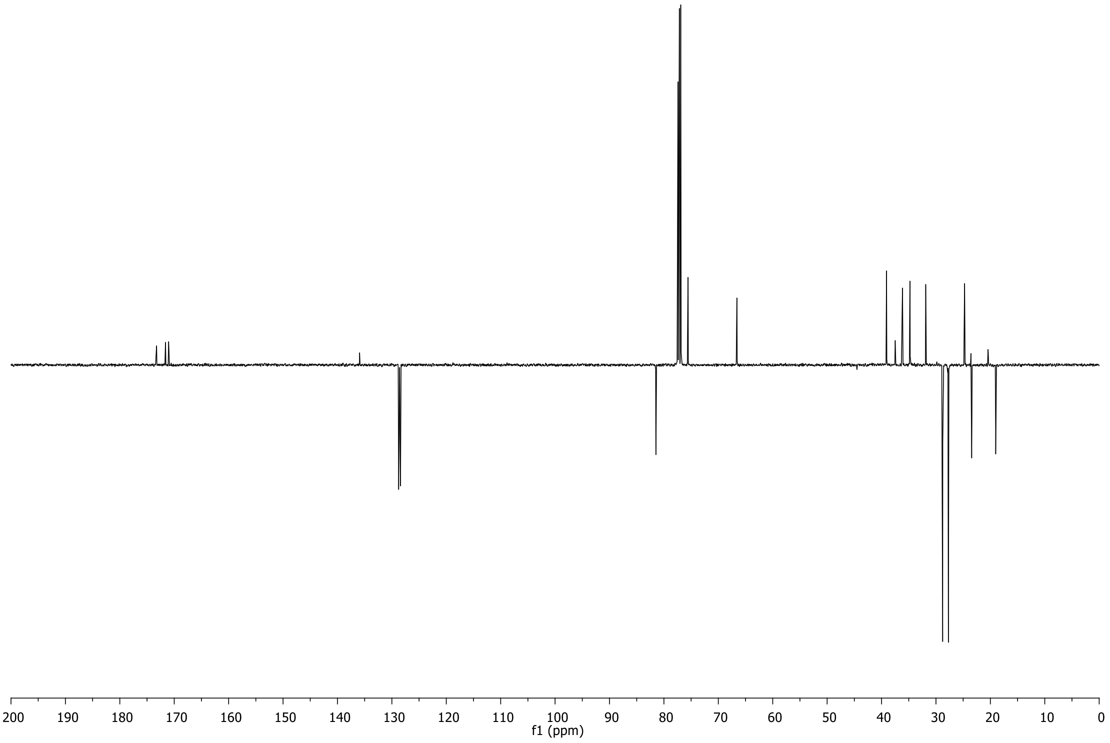

4.2 ^{1}H- and ^{13}C- NMRs of compound 15

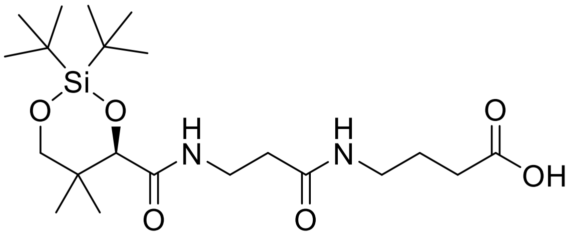

15

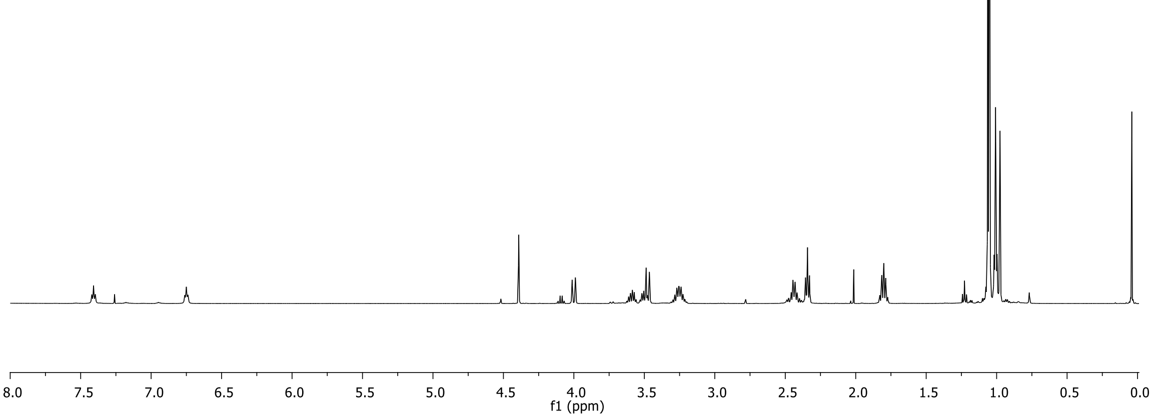

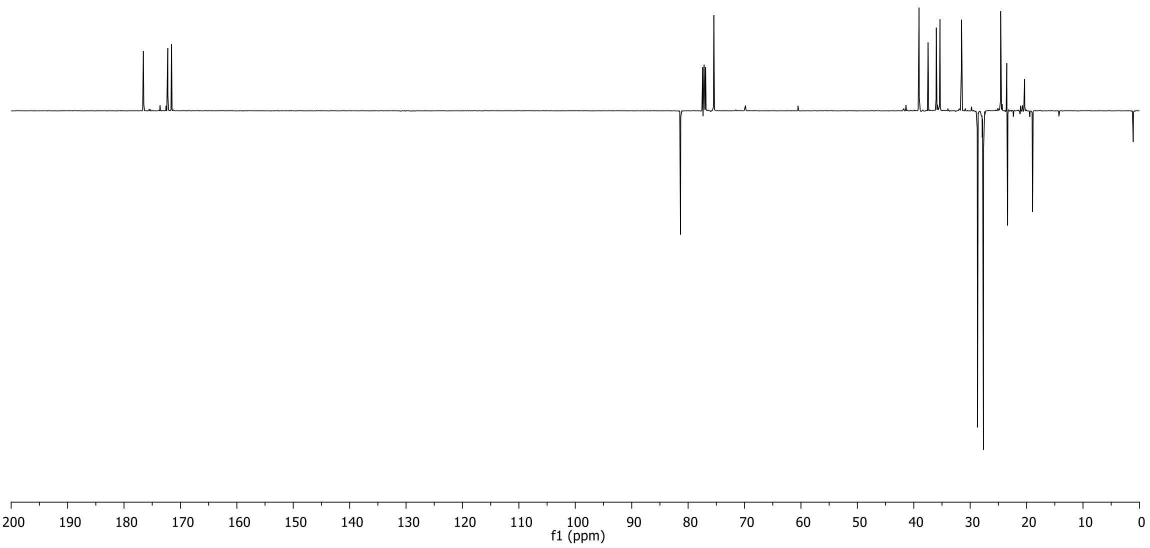

4.3 ¹H- and ¹³C- NMRs of compound 16

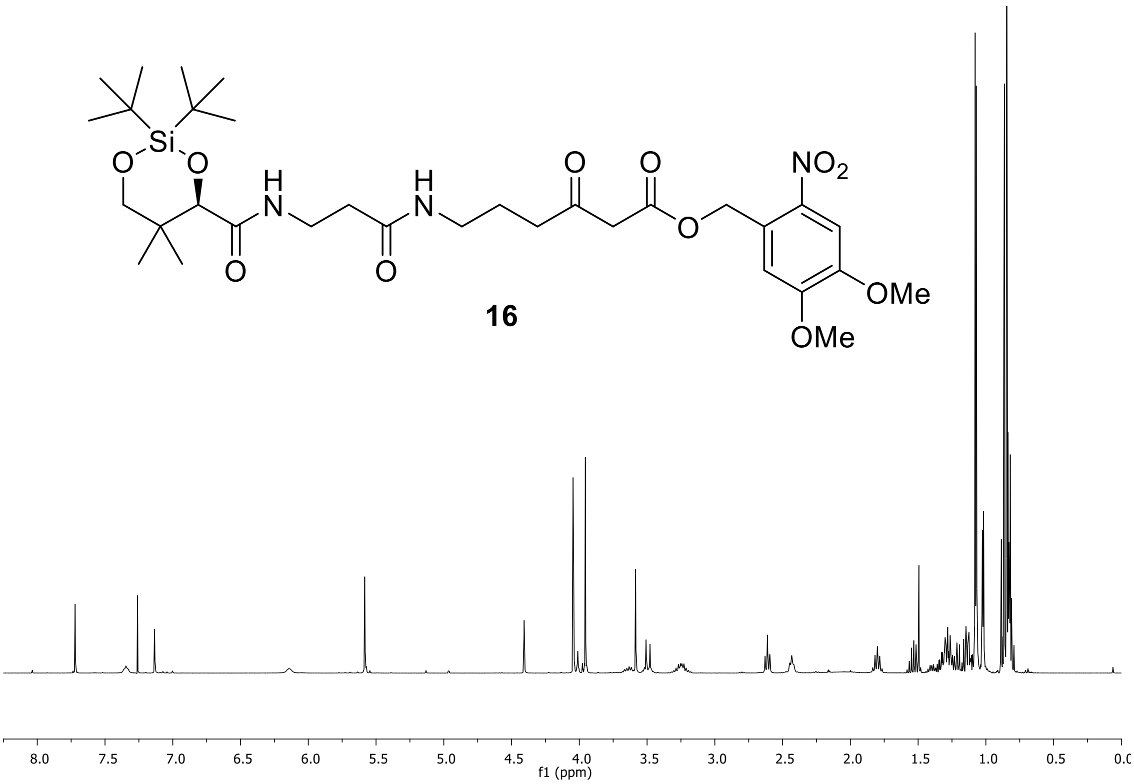

16

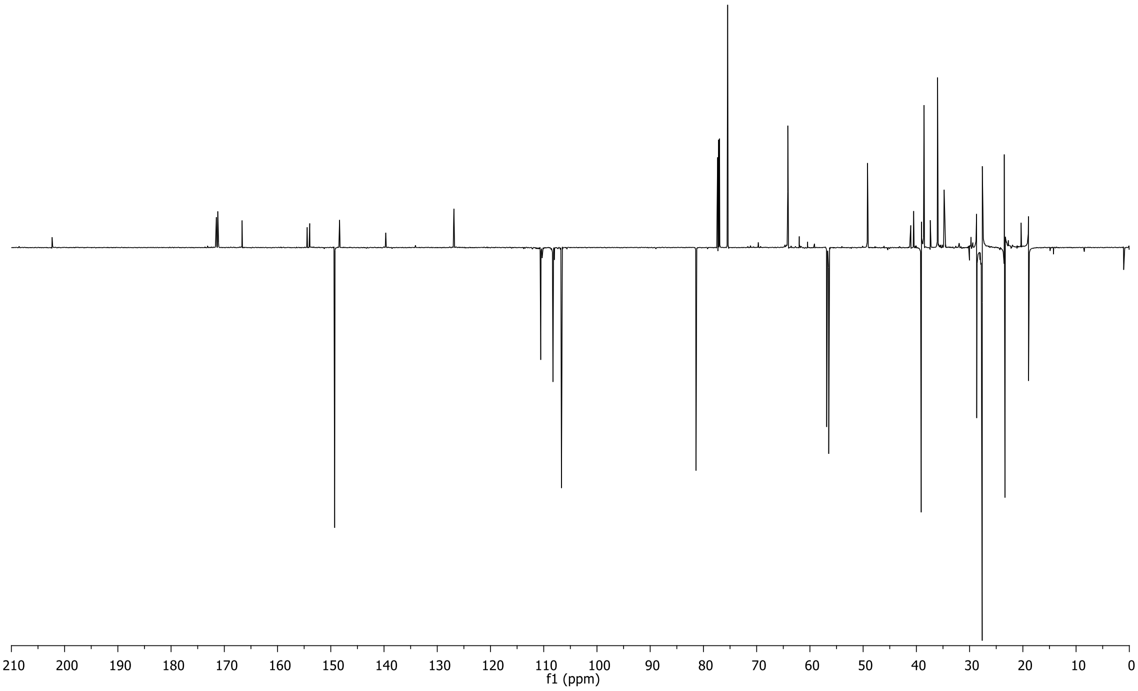

4.4 ¹H- and ¹³C- NMRs of compound 10

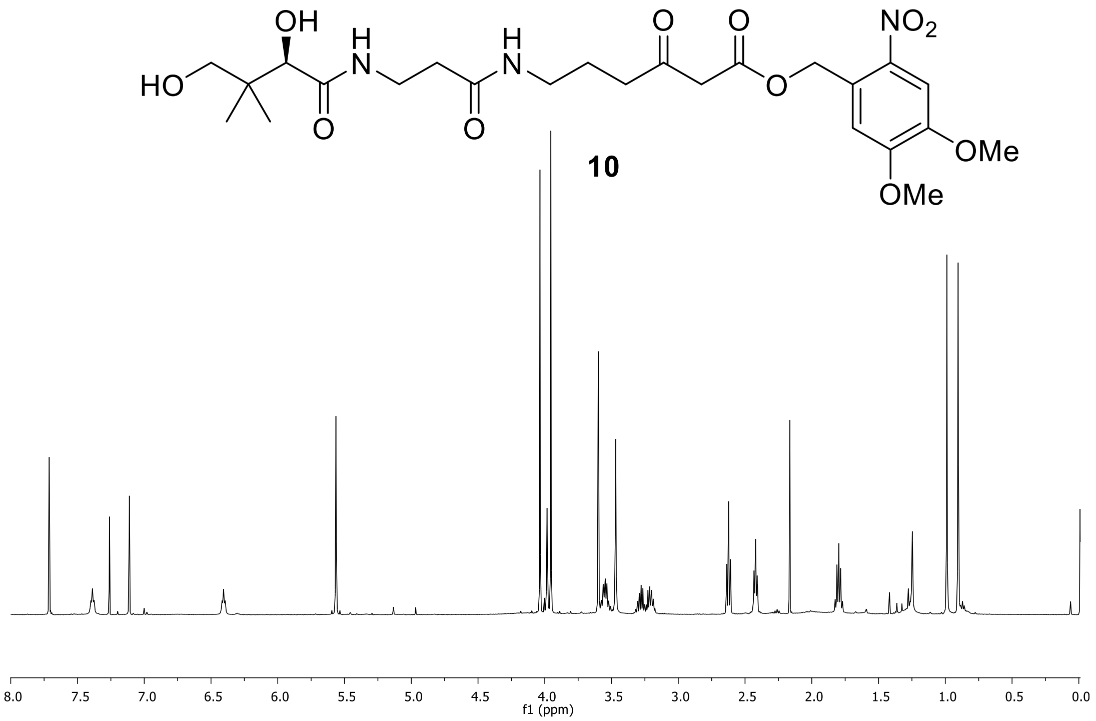

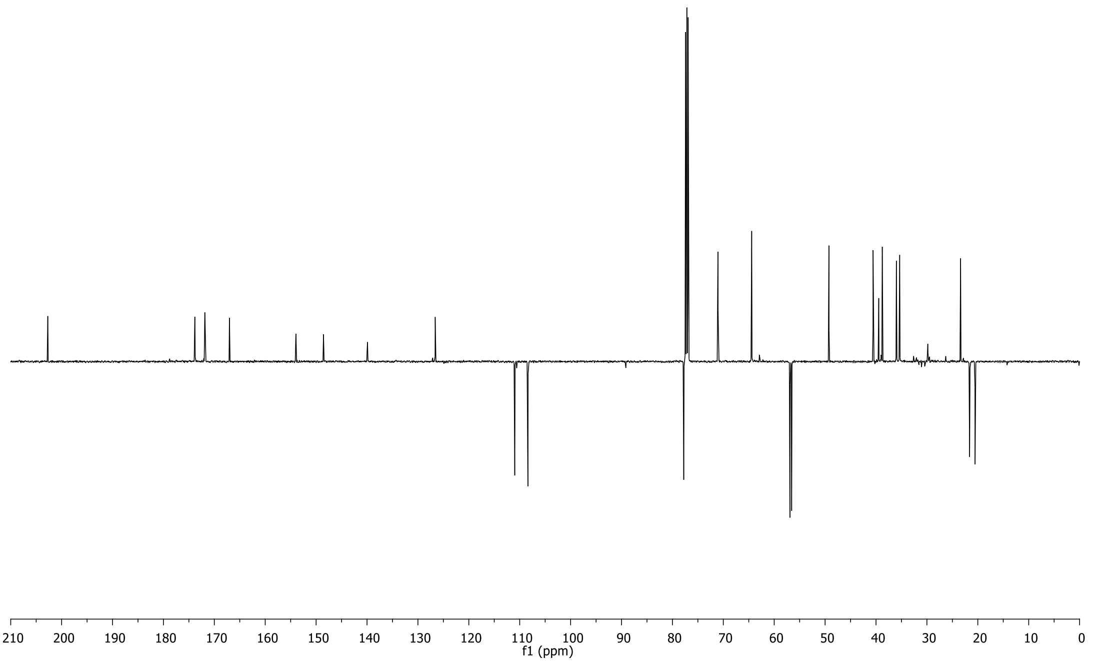

4.5 ¹H- and ¹³C- NMRs of compound 17

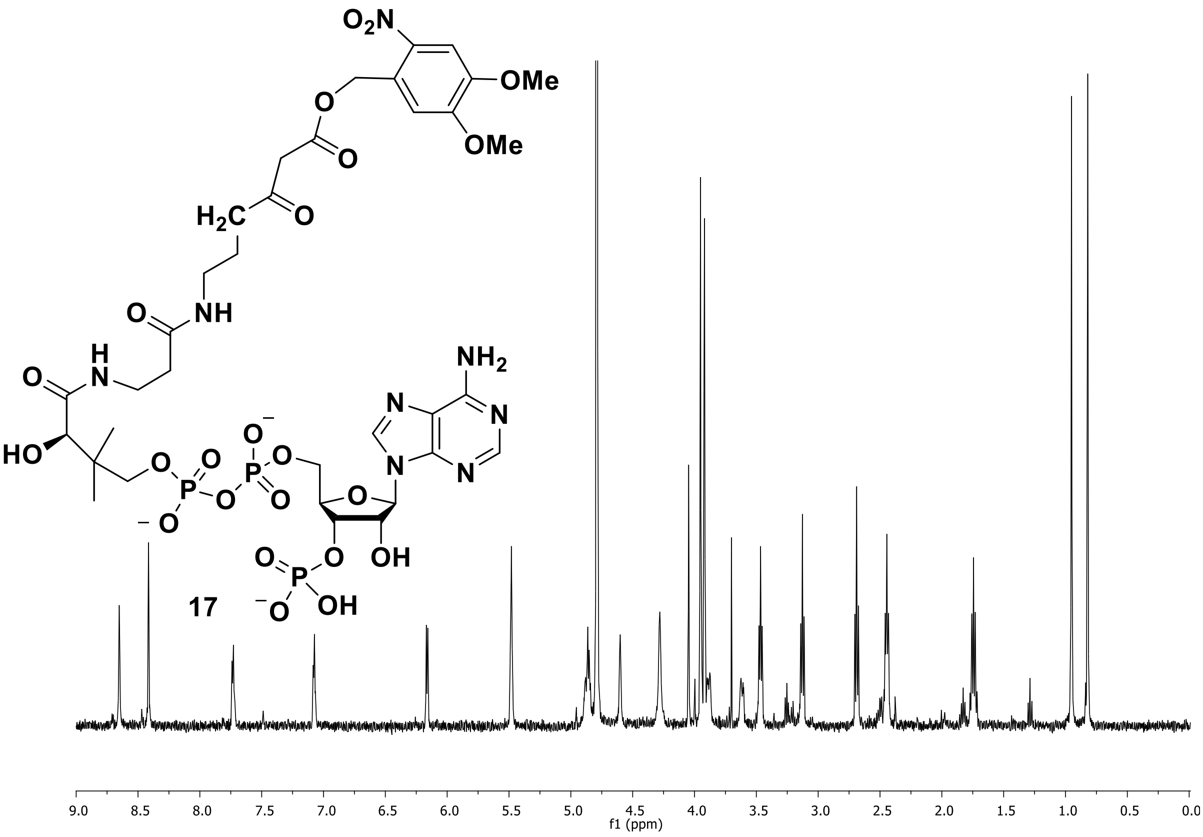

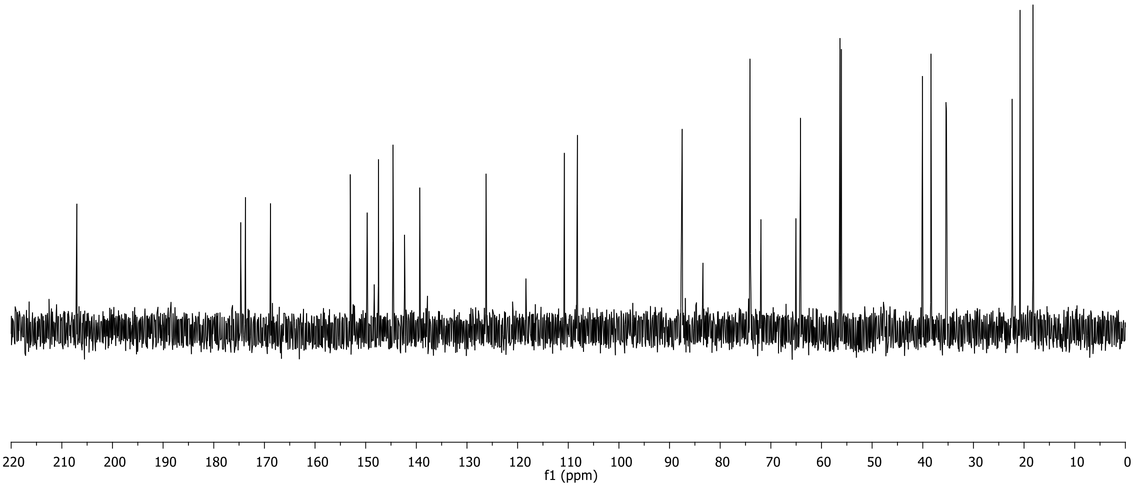

5 References

[1] M. Tosin, D. Spiteller, J. B. Spencer, *ChemBioChem* **2009**, *10*, 1714-1723.

[2] P. C. Dorrestein, S. B. Bumpus, C. T. Calderone, S. Garneau-Tsodikova, Z. D. Aron, P. D. Straight, R. Kolter, C. T. Walsh, N. L.Kelleher, *Biochemistry* **2006**, *45*, 12756-12766

[3] S. L. Kilgour, R. Jenkins, M. Tosin, *Chem. Eur. J.* **2019**, *in review.*

[4] F. Xian, C. L. Hendrickson, G. T. Blakney, S. C. Beu, A. G. Marshall, *Anal. Chem.* **2010**, *82*, 8807–8812.

[5] Y. Qi, H. Li, R. H. Wills, P. Perez-Hurtado, X. Yu, D. P. A. Kilgour, M. P. Barrow, C. Lin, P. B. O'Connor *J. Am. Soc. Mass Spectrom.* **2013**, *24*, 828–834.

[6] D. P. A. Kilgour, R. Wills, Y. Qi, P. B. O'Connor, *Anal. Chem.* **2013**, *85*, 3903–3911.

[7] D. Kilgour, M. J. Neal, A. J. Soulby, P. B. O'Connor, *Rapid Commun. Mass Spectrom.*, **2013**, *27*, 1977–1982.

[8] D. P. A. Kilgour, S. L. Van Orden, *Rapid Commun. Mass Spectrom.* **2015**, *29*, 1009–1018.

[9] D. P. A. Kilgour, S. Hughes, S. L. Kilgour, C. L. Mackay, M. Palmblad, B. Q. Tran, Y. A. Goo, R. K. Ernst, D. J. Clarke, D. R. Goodlett, *J. Am. Soc. Mass Spectrom.* **2017**, *28*, 253–262.

[10] D. P. A. Kilgour, S. L. Van Orden, B. Q. Tran, Y. A. Goo, D. R. Goodlett, *Anal. Chem.* **2015**, *87*, 5797–5801.

[11] A. L. Matharu, R. J. Cox, J. Crosby, K. J. Byrom, T. J. Simpson, *Chem. Biol.* **1998**, *5*, 699-711.

[12] L. E. Quadri, P. H. Weinreb, M. Lei, M. M. Nakano, P. Zuber, C. T. Walsh, *Biochemistry* **1998**, *37*, 1585-1595.
